# Synergistic Electrocatalytic N_2_ Reduction over Asymmetric Heteronuclear Dual Ru‐Fe Sites

**DOI:** 10.1002/advs.202512218

**Published:** 2025-10-14

**Authors:** Zihao Yang, Chao Feng, Yifan Liu, Yong Yang

**Affiliations:** ^1^ College of Biological and Chemical Engineering Qilu Institute of Technology Jinan 250200 China; ^2^ State Key Laboratory of Photoelectric Conversion and Utilization of Solar Energy Qingdao Institute of Bioenergy and Bioprocess Technology Chinese Academy of Sciences Qingdao 266101 China; ^3^ College of Chemical and Biological Engineering Shandong University of Science and Technology Qingdao Shandong 266590 China; ^4^ University of Chinese Academy of Sciences Beijing 100049 China; ^5^ Shandong Energy Institute Qingdao 266101 China

**Keywords:** asymmetric coordination, dual‐atom catalysts, electrocatalytic nitrogen reduction reaction, MXene, synergistic catalysis

## Abstract

The scaling relationship limit poses a significant challenge in single‐atom catalysts (SACs) for reactions involving multi‐intermediate interactions, such as the electrocatalytic nitrogen reduction reaction (eNRR) for ammonia synthesis. To overcome this limitation, a heteronuclear dual Ru‐Fe sites on N,S‐codoped Ti_3_C_2_T_x_ nanosheet (referred to as Fe_1_‐N^S‐Ru_1_/Ti_3_C_2_T_x_) with precisely designed asymmetric coordination for eNRR is developed. Advanced characterizations verify the unique asymmetric coordination structure where Ru and Fe atoms are individually coordinated to N and S atoms, respectively, with the two metal centers interconnected via bridging N and S atoms. This catalyst achieves remarkable eNRR performance with an NH_3_ yield rate of 32.8 µg h^−1^ mg^−1^_cat_ at −0.55 V and 47.1% Faradaic efficiency at −0.25 V, surpassing its homonuclear analogues by 3.2‐ and 2.3‐fold in activity and ≈3.0‐fold in selectivity. Experimental and theoretical studies reveal a synergistic mechanism, in which Ru sites effectively dissociate H_2_O to supply protons while the adjacent Fe sites selectively activate N_2_, effectively decoupling proton supply from N_2_ activation but also benefiting the formation of key intermediate ^*^NNH. Additionally, the electronic interaction between Ru and Fe sites also lowers the energy barrier of the rate‐determining step, thereby significantly enhancing catalytic activity and selectivity.

## Graphical abstract

A heteronuclear Ru‐Fe dual atom catalyst with asymmetryic coordination environment anchored on N,S‐codoped Ti_3_C_2_T_x_ nanosheet is reported. The asymmetric dual Ru‐Fe sites breaks the scaling relationship and synergistically improve eNRR efficiency.

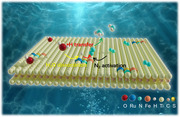

## 1 Introduction

Ammonia (NH_3_) is a chemically pivotal compound with vital applications across agriculture, military defense, and energy storage.^[^ ^1^, ^2^ ^]^ For nearly a century, the industrial NH_3_ production has been dominated by the traditional energy‐intensive Haber‐Bosch process, which operates under extremely harsh conditions (400–600 °C, 150–200 atm).^[^ ^3^ ^]^ This process relies heavily on fossil fuels and contributes to approximately of 2% worldwide natural gas usage and 1% of global CO_2_ emissions.^[^ ^4^, ^5^ ^]^ As a sustainable alternative, the electrocatalytic nitrogen reduction reaction (eNRR) has emerged as a promising pathway for green NH_3_ synthesis from abundant N_2_ and H_2_O at ambient temperature and pressure when powered by renewable electricity.^[^ ^6^ ^]^ This method effectively alleviates environmental concerns and mitigates CO_2_ emissions. However, despite its advantages, eNRR faces two major challenges: i) the inherently sluggish reaction kinetics due to the ultra‐high stability of the N≡N triple bond, and ii) low Faradaic efficiency (FE) caused by the inevitable competing hydrogen evolution reaction (HER) in aqueous electrolytes.^[^ ^7^, ^8^, ^9^, ^10^, ^11^ ^]^ The key to addressing the above challenges lies in developing highly efficient electrocatalysts that can effectively activate N_2_ while simultaneously inhibiting HER.

Atomically dispersed single‐atom catalysts (SACs) have garnered significant attention in the fields of catalysis and energy conversion/storage due to their exceptional atomic utilization efficiency, unique electronic structures, and well‐defined active centers.^[^ ^12^ ^]^ Previous studies have demonstrated that SACs not only exhibit improved catalytic performance but also provide an ideal platform for a better understanding of the structure‐activity relationship at the atomic level.^[^ ^13^, ^14^, ^15^ ^]^ However, when applied to the complex proton‐coupled electron transfer processes of eNRR, conventional SACs face a fundamental dilemma: the uniformly distributed single active sites must simultaneously fulfill two antagonistic functions − activating the N≡N bond while mediating H_2_O dissociation to supply protons. This dual requirement imposes stringent constraints on the active site, necessitating a delicate balance between N_2_ activation and H_2_O dissociation. Compounding this challenge, the intrinsic scaling relationship governing these competing multi‐intermediate interactions inevitably results in compromised catalytic activity and diminished HER suppression.^[^ ^14^ ^]^ Dual‐atom catalysts (DACs) present a promising strategy to overcome the limitation of SACs by integrating two spatially proximate, well‐defined active sites on a localized surface.^[^ ^16^, ^17^, ^18^, ^19^ ^]^ As an extension of SACs, DACs preserve the merits of SACs while introducing additional synergistic benefits through electronic ligand effects and geometric ensemble effects.^[^ ^20^, ^21^ ^]^ These distinctive features have demonstrated extraordinary potential in enabling multifunctional cooperative catalysis, leading to significantly improved performance across various catalytic reactions, including oxygen reduction reaction (ORR), CO_2_ reduction reaction (CO_2_RR), and hydrogen evolution reaction (HER).^[^ ^22^, ^23^, ^24^, ^25^, ^26^, ^27^ ^]^ Surprisingly, despite their potential, the exploration of DACs for the challenging eNRR remains largely underdeveloped. To address this critical gap and meet the pressing need for efficient bifunctional catalysts capable of simultaneously activating inert N_2_ and mediating H_2_O dissociation during eNRR, we propose an innovative DAC architecture featuring two functionally specialized active sites. This novel design paradigm strategically circumvents the inherent limitation of conventional SACs by fostering cooperative interactions between the two adjacent sites, thereby creating synergistic catalysis that could potentially unlock unprecedented eNRR performance.

Keeping the above consideration in mind, in this work, we design a novel heteronuclear dual Ru‐Fe sites with an asymmetric configuration anchored on sulfur‐ and nitrogen‐codoped Ti_3_C_2_T_x_ MXene for eNRR. In this unique architecture, Ru and Fe atoms are selectively coordinated to N and S atoms, respectively, with the two metal centers interconnected through bridging N and S atoms, denoted as Fe_1_‐N^S‐Ru_1_/Ti_3_C_2_T_x_. Comprehensive characterizations, including aberration‐corrected high‐angle annular dark‐field scanning transmission electron microscopy (HAADF‐STEM), X‐ray photoelectron spectroscopy (XPS), extended X‐ray absorption near‐edge structure (XANES) spectroscopy, and extended X‐ray absorption fine structure (EXAFS) spectroscopy, confirmed the precise atomic configuration and electronic structure of the catalyst. The Fe_1_‐N^S‐Ru_1_/Ti_3_C_2_T_x_ catalyst exhibited exceptional eNRR performance, delivering an NH_3_ yield rate of 32.8 µg h^−1^ mg^−1^_cat_ at −0.55 V versus RHE and a Faradaic efficiency (FE) of 47.1% at −0.25 V versus RHE, respectively, significantly outperforming its homonuclear counterparts. Notably, the NH_3_ yield rate was 3.2‐fold and 2.3‐fold higher than those of Ru_1_‐N^S‐Ru_1_/Ti_3_C_2_T_x_ and Fe_1_‐N^S‐Fe_1_/Ti_3_C_2_T_x_, respectively, while the FEs similarly exhibited ≈3.0‐fold enhancement compared to both symmetric configurations. Mechanistic insights from experimental and theoretical calculations revealed that the asymmetric dual Ru‐Fe sites facilitate unidirectional electron transfer between Ru and Fe atoms and enable synergistic catalysis, in which the Ru sites preferentially dissociate H_2_O to produce reactive H species, while the adjacent Fe sites efficiently activate N_2_ molecules. This cooperative mechanism effectively decouples the competing requirement of proton supply and N_2_ activation, while simultaneously lowering the energy barrier of the potential rate‐determining step, thereby substantially boosting catalytic efficiency.

## 2 Results and Discussion

**Figure 1:**
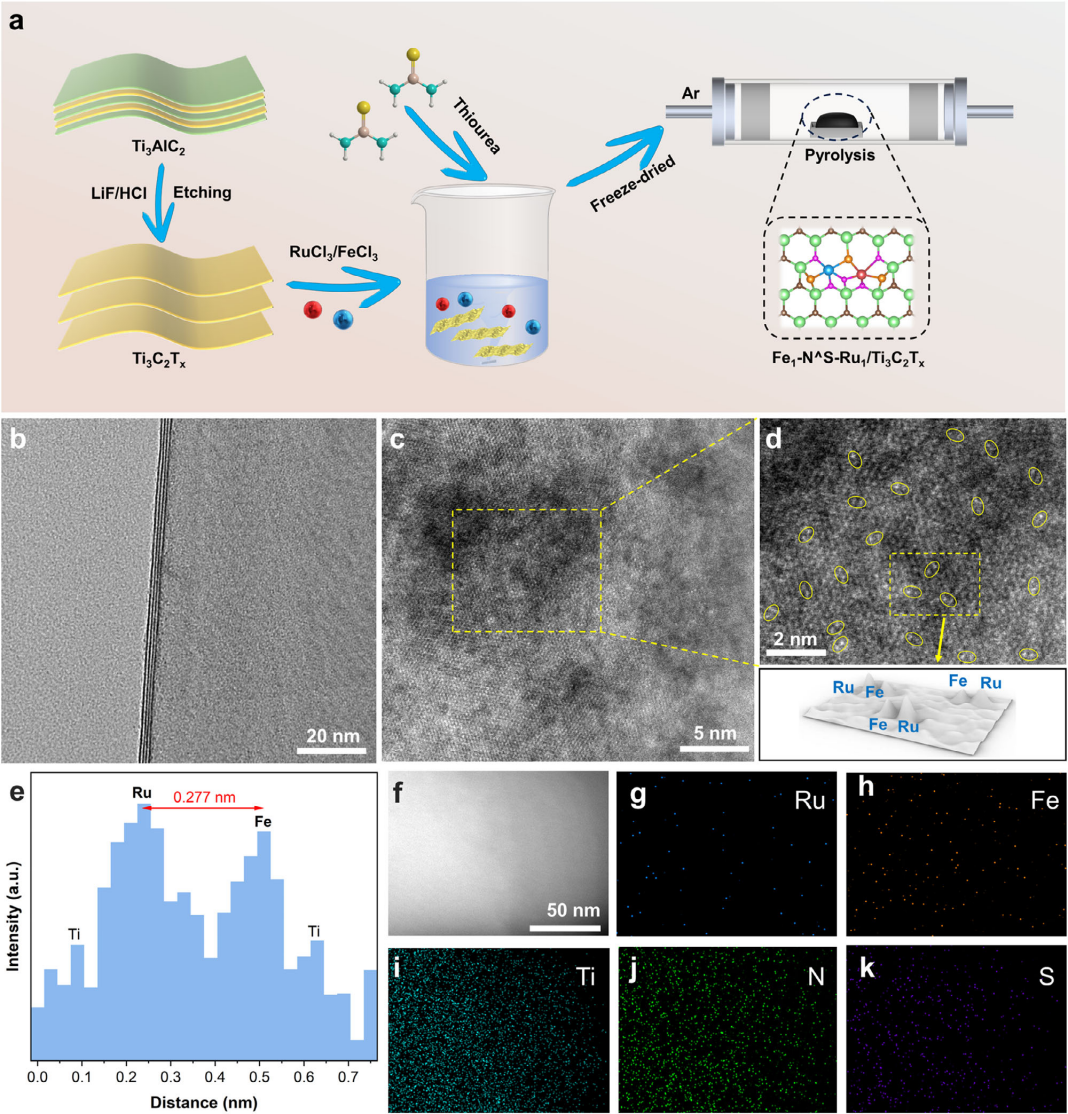
a) Schematic illustration for preparing the electrocatalyst Fe_1_‐N^S‐Ru_1_/Ti_3_C_2_T_x_. b) HR‐TEM image of Fe_1_‐N^S‐Ru_1_/Ti_3_C_2_T_x_, c) AC HAADF‐STEM image of Fe_1_‐N^S‐Ru_1_/Ti_3_C_2_T_x_, d) Enlarged image and 3D intensity surface plot of Fe_1_‐N^S‐Ru_1_/Ti_3_C_2_T_x_, e) Distance between two metal sites, f‐k) EDS mappings of Fe_1_‐N^S‐Ru_1_/Ti_3_C_2_T_x_ and the distribution of Ru, Fe, Ti, N, and S elements.

**Figure** **1**a schematically illustrates the synthesis procedure for the asymmetric heteronuclear dual Fe‐Ru sites co‐bridged by N and S atoms on monolayer Ti_3_C_2_T_x_ nanosheets, referred to as Fe_1_‐N^S‐Ru_1_/Ti_3_C_2_T_x_. The monolayer Ti_3_C_2_T_x_ nanosheets were prepared according to our previous work.^[^ ^28^, ^29^ ^]^ Briefly, a specific amount of Fe and Ru precursors was thoroughly mixed with urea in an aqueous solvent, which was then added to the aqueous dispersion of monolayer Ti_3_C_2_T_x_ nanosheets under vigorous stirring. The resulting mixture was subjected to freeze‐drying to produce a 3D Ti_3_C_2_T_x_ aerogel. The direct pyrolysis of the aerogel at 500 °C for 2 h in an Ar atmosphere transformed urea into N, S‐co‐incorporated Ti_3_C_2_T_x_ while concurrently trapping the Fe and Ru species. For comparative purposes, DACs with symmetric homonuclear dual Ru or Fe sites co‐bridged by N and S atoms on monolayer Ti_3_C_2_T_x_ nanosheets were also synthesized using the same procedure, omitting the introduction of the second metal precursor in each case, which are labeled as Ru_1_‐N^S‐Ru_1_/Ti_3_C_2_T_x_ and Fe_1_‐N^S‐Fe_1_/Ti_3_C_2_T_x_, respectively. The weight loading of Fe and Ru in Fe_1_‐N^S‐Ru_1_/Ti_3_C_2_T_x_ was measured to be 1.43 and 1.56%, 2.95% of Ru in Ru_1_‐N^S‐Ru_1_/Ti_3_C_2_T_x_, and 2.84% of Fe in Fe_1_‐N^S‐Fe_1_/Ti_3_C_2_T_x_ (Table S1, Supporting Information), respectively, as determined by inductively coupled plasma atomic emission spectroscopy (ICP‐AES). In addition, N and S atoms co‐doped monolayer Ti_3_C_2_T_x_ nanosheets, named as N, S‐Ti_3_C_2_T_x_, were also prepared.

Scanning electron microscopy (SEM) image (Figure S1, Supporting Information), high‐resolution transmission electron microscopy (HR TEM) (Figure 1b; Figure S2, Supporting Information), and selected area electron diffraction (SAED) images (Figure S3, Supporting Information) of Fe_1_‐N^S‐Ru_1_/Ti_3_C_2_T_x_ reveal that the monolayered structure of Ti_3_C_2_T_x_ nanosheets was well‐preserved after the incorporation of heteronuclear dual Ru‐Fe sites, with no observation of metal nanoparticles and/or nanoclusters. The X‐ray diffraction (XRD) patterns (Figure S4, Supporting Information) of Fe_1_‐N^S‐Ru_1_/Ti_3_C_2_T_x_ only show several characteristic diffraction peaks corresponding to the pristine Ti_3_C_2_T_x_. However, the characteristic (002) and (004) diffraction peaks exhibit a slight positive shift relative to those of the pristine Ti_3_C_2_T_x_, suggesting an increased interlayer spacing stemming from the intercalation of heteroatoms (N, S, Fe, and Ru) during the pyrolysis process.^[^ ^30^ ^]^ The aberration‐corrected high‐angle annular dark‐field scanning transmission electron microscopy (AC HAADF‐STEM) image (Figure 1c) shows numerous isolated bright spots throughout the Ti_3_C_2_T_x_ surface in the Fe_1_‐N^S‐Ru_1_/Ti_3_C_2_T_x_, indicating an atomic dispersion of Fe and Ru sites. An enlarged AC HAADF‐STEM image (Figure 1d) clearly distinguishes that most of the bright spots exist in paired configurations, as shown in the 3D atom‐overlapping Gaussian‐function fitting map (Figure 1e). Statistical analysis of the dual‐atom pairs (Figure 1f) reveals that the distance between paired Ru and Fe atoms is 0.277 nm, which is significantly longer than the typical metal‐metal bond length (≈0.23 nm) in metals.^[^ ^31^ ^]^ This observation suggests that the Fe and Ru atoms are likely bridged by N and S atoms rather than directly bonding to each other.^[^ ^22^, ^32^ ^]^ In addition, the energy dispersive spectroscopy (EDS) mapping of Fe_1_‐N^S‐Ru_1_/Ti_3_C_2_T_x_ (Figure 1g–l) shows a uniform distribution of Ru, Fe, N, and S elements across the catalyst surface.

**Figure 2:**
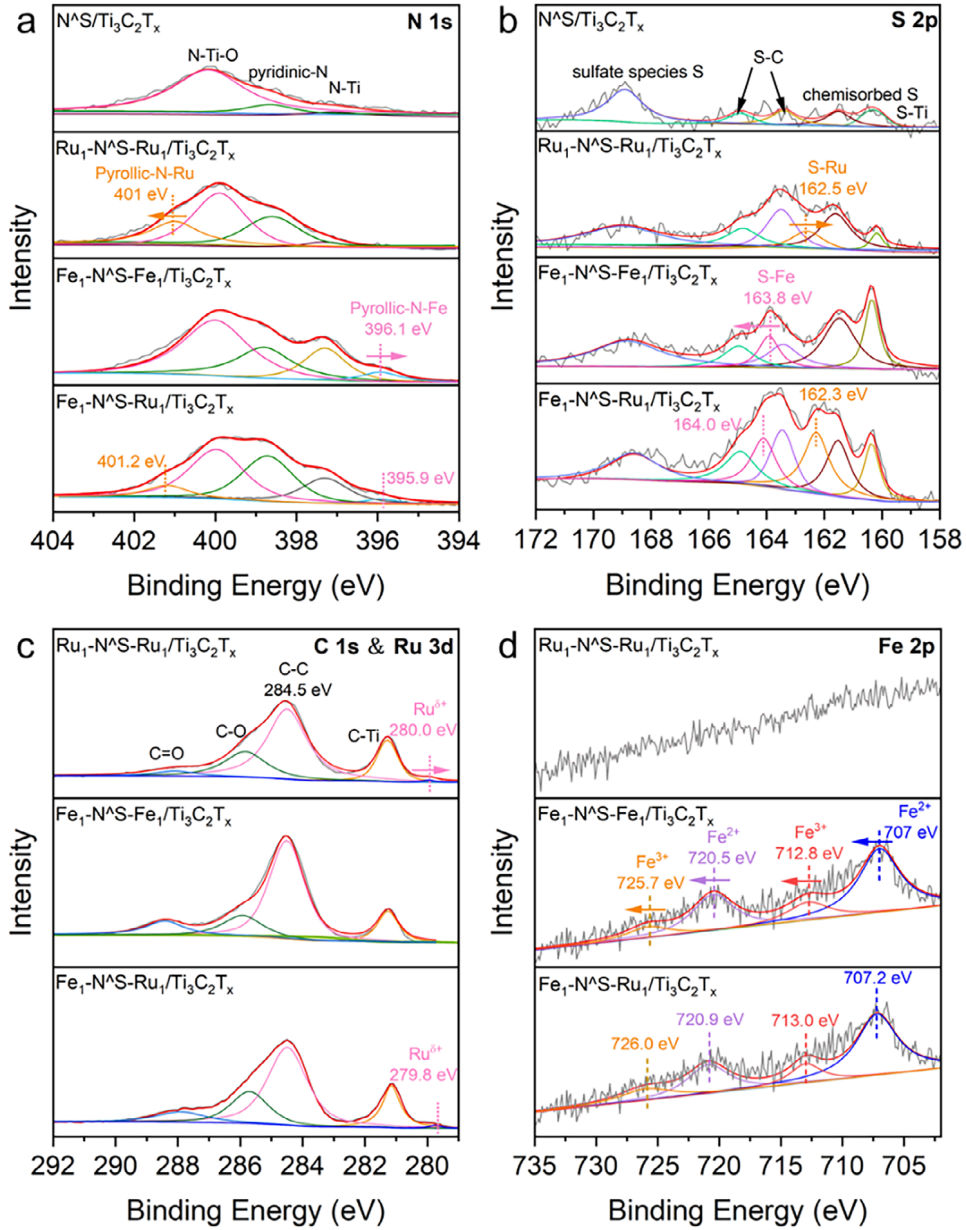
XPS analysis of a) N 1s, b) S 2p, c) C 1s, Ru 3d and d) Fe 2p for Ru_1_‐N^S‐Ru_1_/Ti_3_C_2_T_x_, Fe_1_‐N^S‐Fe_1_/Ti_3_C_2_T_x_, Fe_1_‐N^S‐Ru_1_/Ti_3_C_2_T_x_, and N^S/Ti_3_C_2_T_x_.

X‐ray photoelectron spectroscopy (XPS) was then employed to investigate the surface composition and chemical states of Fe_1_‐N^S‐Ru_1_/Ti_3_C_2_T_x_. The N 1s spectrum of Fe_1_‐N^S‐Ru_1_/Ti_3_C_2_T_x_ (**Figure** **2**a) exhibits five distinct peaks. Among these, three distinct peaks at 397.2 (Ti‐N), 398.7 (pyridinic N), and 400.5 eV (N‐Ti‐O) originate from the N^S/Ti_3_C_2_T_x_.^[^ ^33^ ^]^ Two additional peaks at 395.9 and 401.2 eV were observed, assigned to Fe‐N and Ru‐N species,^[^ ^34^, ^35^ ^]^ respectively, confirming the successful formation of Fe─N and Ru─N bonds in Fe_1_‐N^S‐Ru_1_/Ti_3_C_2_T_x_. In comparative analysis, Ru_1_‐N^S‐Ru_1_/Ti_3_C_2_T_x_ exhibits only a Ru─N bond peak at 401.0 eV, while Fe_1_‐N^S‐Fe_1_/Ti_3_C_2_T_x_ displays a single Fe─N bond peak at 396.1 eV. Notably, there is a 0.2 eV positive shift in the binding energy of Ru─N species, coupled with a 0.2 eV negative shift in the binding energy of Fe─N species in the heteronuclear Fe_1_‐N^S‐Ru_1_/Ti_3_C_2_T_x_ relative to their homonuclear counterparts, suggesting an electronic interaction between the Ru and Fe sites. The S 2p spectrum of Fe_1_‐N^S‐Ru_1_/Ti_3_C_2_T_x_ (Figure 2b) was deconvoluted into six distinct peaks. Of them, two doublets with a characteristic 2:1 area ratio and 1.2 eV spin‐orbit splitting are identified at 160.2/161.4 eV and 163.2/164.4 eV, corresponding to Ti─S and C─S─C bond in 2p_3/2_ and 2p_1/2_ orbitals, respectively.^[^ ^33^, ^36^ ^]^ An additional peak at 168.5 eV indicates the presence of oxidized S, including ─SO_2_ groups. Furthermore, two new peaks at 162.3 and 164.0 eV, corresponding to Ru─S and Fe─S bonds,^[^ ^33^, ^37^ ^]^ were observed in Fe_1_‐N^S‐Ru_1_/Ti_3_C_2_T_x_. In contrast, Ru_1_‐N^S‐Ru_1_/Ti_3_C_2_T_x_ shows a single peak at 162.5 eV, and Fe_1_‐N^S‐Fe_1_/Ti_3_C_2_T_x_ displays a peak at 163.8 eV. Additionally, a 0.2 eV shift in the binding energies of the Ru─S and Fe─S bonds in Fe_1_‐N^S‐Ru_1_/Ti_3_C_2_T_x_ relative to the homonuclear Ru_1_‐N^S‐Ru_1_/Ti_3_C_2_T_x_ and Fe_1_‐N^S‐Fe_1_/Ti_3_C_2_T_x_ supports the electronic interactions between the Ru and Fe sites, consistent with observations in the N 1s XPS. In the Ru 3d spectra (Figure 2c), a low‐intensity peak at 279.8 eV was observed for Fe_1_‐N^S‐Ru_1_/Ti_3_C_2_T_x_, while a peak at 280.0 eV was detected for Ru_1_‐N^S‐Ru_1_/Ti_3_C_2_T_x_.^[^ ^38^, ^39^ ^]^ No peak at this position was observed for Fe_1_‐N^S‐Fe_1_/Ti_3_C_2_T_x_. In the Fe 2p spectra (Figure 2d), two sets of peaks at 707.0 and 712.8 eV were identified for Fe_1_‐N^S‐Fe_1_/Ti_3_C_2_T_x_ and Fe_1_‐N^S‐Ru_1_/Ti_3_C_2_T_x_, corresponding to Fe^2+^ and Fe^3+^ species, respectively.^[^ ^40^, ^41^ ^]^ A 0.2 eV shift in the binding energy for both Ru 3d and Fe 2p was also noticed when comparing the heteronuclear dual Ru‐Fe sites with homonuclear Ru‐Ru or Fe‐Fe sites, further confirming the electronic interaction between Fe and Ru sites within Fe_1_‐N^S‐Ru_1_/Ti_3_C_2_T_x_.

**Figure 3:**
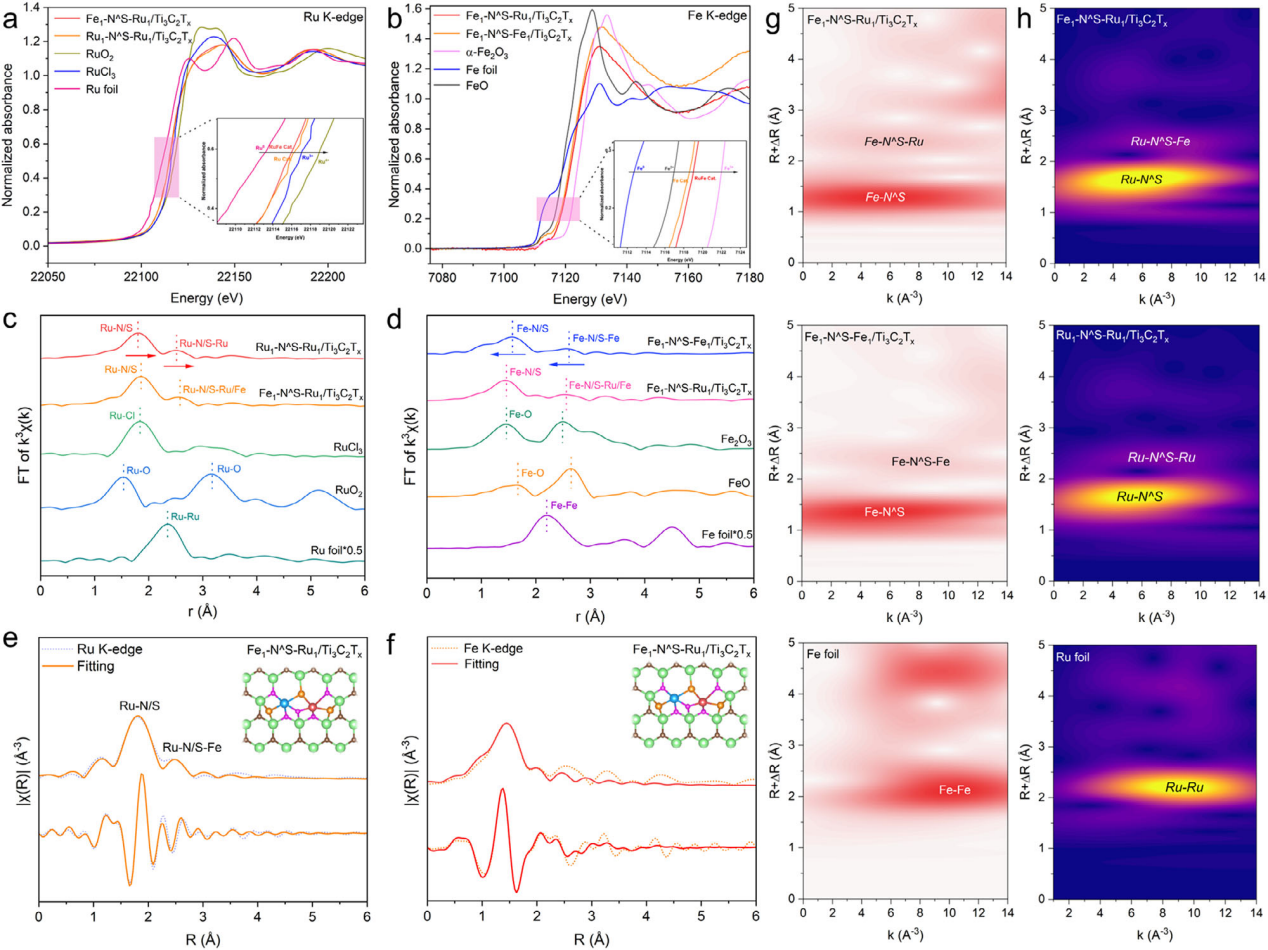
XANES spectra for a) Ru K‐edge and b) Fe K‐edge. Fourier transformed k^3^‐weighted EXAFS spectra for c) Ru K‐edge and d) Fe edge Fe_1_‐N^S‐Ru_1_/Ti_3_C_2_T_x_, Ru_1_‐N^S‐Ru_1_/Ti_3_C_2_T_x,_ and Fe_1_‐N^S‐Fe_1_/Ti_3_C_2_T_x_ with the references. e) EXAFS R‐space curve‐fitting curve of Fe_1_‐N^S‐Ru_1_/Ti_3_C_2_T_x_ at e) Ru K‐edge and f) Fe K‐edge. The inset represents the atomic coordination fitting model. In the inset, the blue, red, brown, green, pink, and orange spheres represent Ru, Fe, Ti, C, N, and O elements, respectively. WT contour plots of g) Fe K‐edge and h) Ru K‐edge for Fe_1_‐N^S‐Ru_1_/Ti_3_C_2_T_x_, Fe foil, and Ru foil.

To further elucidate the atomic structure and coordination environment of the Fe and Ru species, synchrotron radiation X‐ray absorption near edge structure (XANES) and extended X‐ray absorption fine structure (EXAFS) spectra were collected for Fe_1_‐N^S‐Ru_1_/Ti_3_C_2_T_x_, Ru_1_‐N^S‐Ru_1_/Ti_3_C_2_T_x_, and Fe_1_‐N^S‐Fe_1_/Ti_3_C_2_T_x_, alongside Ru foil, RuO_2_, RuCl_3_, Fe foil, FeO, and Fe_2_O_3_ as the references. As shown in **Figure** **3**a, the Ru absorption edge of Fe_1_‐N^S‐Ru_1_/Ti_3_C_2_T_x_ lies between those of Ru foil and RuCl_3,_ closer to RuCl_3_, indicating that the valence state of the Ru species is ≈+3. Compared to the homonuclear Ru_1_‐N^S‐Ru_1_/Ti_3_C_2_T_x_, the Ru absorption edge of heteronuclear Fe_1_‐N^S‐Ru_1_/Ti_3_C_2_T_x_ exhibits a slight negative shift, suggesting a reduced valence state due to electronic interaction between Ru and Fe sites with electron transfer from Fe to Ru atoms. The Fe absorption edge position (Figure 3b) of Fe_1_‐N^S‐Ru_1_/Ti_3_C_2_T_x_ is located between those of FeO and Fe_2_O_3_, indicating a valence state ranging from +2 to +3. Moreover, the edge position of Fe_1_‐N^S‐Ru_1_/Ti_3_C_2_T_x_ exhibits a positive shift relative to that of the homonuclear Fe_1_‐N^S‐Fe_1_/Ti_3_C_2_T_x_, in contrast to the shift observed in the Ru absorption edge position. This opposite shift provides evidence of electronic interactions between Fe and Ru sites in Fe_1_‐N^S‐Ru_1_/Ti_3_C_2_T_x_, involving electron transfer from Fe to Ru sites, consistent with the XPS result.

The Fourier‐transformed (FT) k^3^‐weighted EXAFS oscillation of Fe_1_‐N^S‐Ru_1_/Ti_3_C_2_T_x_ in *R* space (Figure 3c) presents a prominent peak at approximately 1.80 Å, primarily attributed to the scattering of the Ru‐N and Ru‐S within the first coordination shell.^[^ ^42^ ^]^ A less intense peak at 2.60 Å was also observed, corresponding to the paired Fe‐Ru scattering path. The Fe─Ru bond distance is notably longer than that of the directly coordinated Fe─Ru bonds (2.30 Å), suggesting that this scattering arises from metal coordination in the second shell. Notably, no characteristic scattering peaks for Ru–Ru (2.34 Å) or Ru─O─Ru (3.20 Å) coordination were observed in either Fe_1_‐N^S‐Ru_1_/Ti_3_C_2_T_x_ or Ru_1_‐N^S‐Ru_1_/Ti_3_C_2_T_x_ when compared to Ru foil and RuO_2_, ruling out the presence of metallic Ru or RuO_x_ species. This finding aligns with the HRTEM and HAADF‐STEM results. The Fe K‐edge EXAFS spectra of Fe_1_‐N^S‐Ru_1_/Ti_3_C_2_T_x_ (Figure 3d) exhibit a prominent peak at 1.80 Å and a low‐intensity peak at 2.52 Å, which correspond to the Fe‐N and Fe‐S scattering in the first shell as well as Fe‐Ru scattering in the second shell, respectively.^[^ ^43^ ^]^ Additionally, no scattering peaks associated with Fe‐Fe or Fe‐O‐Fe bonds were observed in either Fe_1_‐N^S‐Ru_1_/Ti_3_C_2_T_x_ or Fe_1_‐N^S‐Fe_1_/Ti_3_C_2_T_x_, confirming the atomic dispersion of Fe sites. Furthermore, the peak positions for both Ru‐N/S and Ru‐Fe scattering paths in the heteronuclear Fe_1_‐N^S‐Ru_1_/Ti_3_C_2_T_x_ slightly shift to higher values compared to those in the homonuclear Ru_1_‐N^S‐Ru_1_/Ti_3_C_2_T_x_. In contrast, the peak positions for both Fe‐N/S and Ru‐Fe scattering in the heteronuclear Fe_1_‐N^S‐Ru_1_/Ti_3_C_2_T_x_ show a negative shift compared to those in the homonuclear Fe_1_‐N^S‐Fe_1_/Ti_3_C_2_T_x_. This observation indicates a local electron redistribution between Fe and Ru sites in heteronuclear Fe_1_‐N^S‐Ru_1_/Ti_3_C_2_T_x_, resulting in changes in bond distances and further supporting the presence of scattering signals arising from mutual interference between Fe and Ru atoms. These findings provide evidence of the unique coordination environment and electronic interactions within heteronuclear Fe_1_‐N^S‐Ru_1_/Ti_3_C_2_T_x_.

Quantitative EXAFS fitting was further performed in the *R* and *k* space to elucidate the local coordination configuration of Fe and Ru in Fe_1_‐N^S‐Ru_1_/Ti_3_C_2_T_x_ (Figure 3e,f), with the extracted structural parameters listed in Table S2 (Supporting Information). The average coordination numbers (CN) of the first shell for Ru (Fe)‐N and Ru (Fe)‐S path are determined to be 3.2 (3.1) and 1.9 (1.9), respectively, indicating that each Ru (or Fe) atom preferentially bonds with three N atoms and two S atoms to form Ru(Fe)N_3_S_2_ structure. To further validate the possible structures of the heteronuclear Fe_1_‐N^S‐Ru_1_/Ti_3_C_2_T_x_, density functional theory (DFT) calculations were performed, which demonstrated that an asymmetric configuration is more stable. In this configuration, the Ru (Fe) atom bonds with two N atoms and one S atom while co‐bridging with one N and one S atom to form the N_2_S‐Ru‐N^˄^S‐Fe‐N_2_S ensemble. Furthermore, based on this calculated structural model, a comparison between the simulated EXAFS spectra of the proposed structures and the experimental spectra was made. As illustrated in Figure 3e,f, the simulated spectra for the N_2_S‐Ru‐N^˄^S‐Fe‐N_2_S ensemble closely aligned well with the experimental results, yielding an *R‐*factor below 0.02. This correlation thereby validates the rationale of the proposed model. Consequently, the N and S co‐bridged heteronuclear dual Ru and Fe active sites featuring an asymmetric configuration are likely representative of the actual local coordination structure in Fe_1_‐N^S‐Ru_1_/Ti_3_C_2_T_x_. For comparison, homonuclear Ru_1_‐N^S‐Ru_1_/Ti_3_C_2_T_x_ and Fe_1_‐N^S‐Fe_1_/Ti_3_C_2_T_x_ with dual Ru and Fe active sites were also constructed, which exhibited symmetric configurations of N_2_S‐Ru‐N^˄^S‐Ru‐N_2_S and N_2_S‐Fe‐N^˄^S‐Fe‐N_2_S, respectively (Figures S5 and S6, Supporting Information).

The wavelet transform (WT) contour plot provides an intuitive validation of the coordination environment of Ru and Fe in Fe_1_‐N^S‐Ru_1_/Ti_3_C_2_T_x_. As illustrated in Figure 3g,h, the WT profile of Ru K‐edge exhibits a high‐intensity region at ≈5.5 Å^−1^ in *k* space and 1.7 Å in *R* space, alongside a less intense region at ≈8.0 Å^−1^ in *k* space and 2.5 Å in *R* space. These features correspond to Ru─N/S bonds and Ru─N^S─Fe bridged bonds, respectively. Similarly, the WT contour plot of Fe K‐edge shows a maximum intensity at ≈5.1Å^−1^ in *k* space and 1.4 Å in *R* space along with a less intense region at ≈6.3 Å^−1^ in *k* space and 2.5 Å in *R* space, which are attributed to the Fe─N/S bonds and Ru─N^S─Fe bridged bonds, respectively. Notably, the overall contour map does not display any signal peaks corresponding to Ru‐Ru or Fe‐Fe sites when compared to Ru/Fe foil, RuO_2_, and FeO_x_ (Figures S7 and S8, Supporting Information), further confirming the atomic dispersion of Fe and Ru in Fe_1_‐N^S‐Ru_1_/Ti_3_C_2_T_x_.

**Figure 4:**
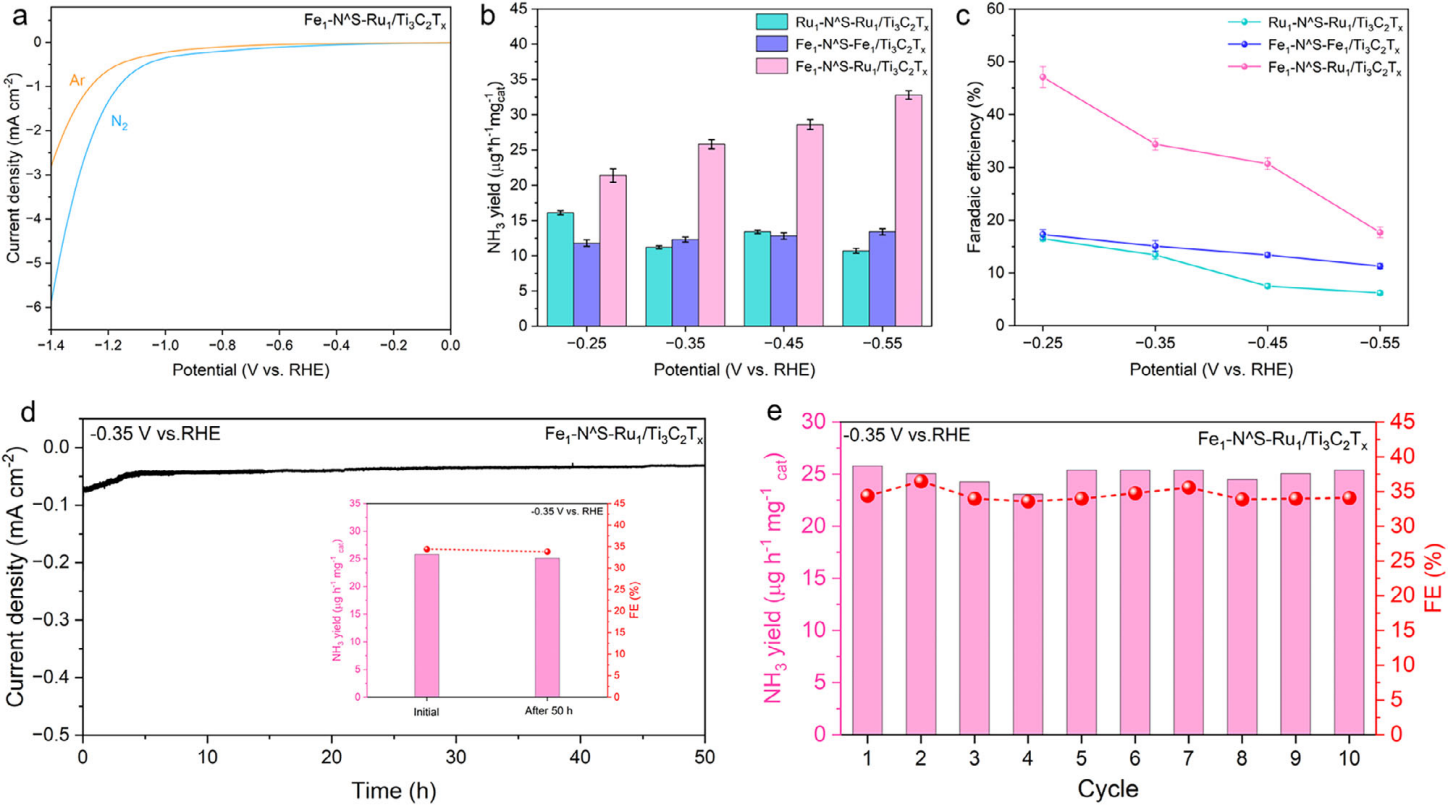
a) LSV curves of Fe_1_‐N^S‐Ru_1_/Ti_3_C_2_T_x_ in aqueous 0.1 m Na_2_SO_4_ solution of Ar‐ and N_2_‐ atmospheres. b) NH_3_ yield rates and c) FEs for Fe in Ru_1_‐N^S‐Ru_1_/Ti_3_C_2_T_x_, Fe in Fe_1_‐N^S‐Fe_1_/Ti_3_C_2_T_x_ and Fe_1_‐N^S‐Ru_1_/Ti_3_C_2_T_x_. d) CA test of Fe_1_‐N^S‐Ru_1_/Ti_3_C_2_T_x_ for 50 h electrolysis at −0.35 V versus RHE, d inset) NH_3_ yield and FEs before and after 50 h CA test. e) Cycling tests of Fe_1_‐N^S‐Ru_1_/Ti_3_C_2_T_x_ for ten cycles of CA runs at −0.35 V versus RHE.

To examine the electrocatalytic nitrogen reduction reaction (eNRR) performance of Fe_1_‐N^S‐Ru_1_/Ti_3_C_2_T_x_, Ru_1_‐N^S‐Ru_1_/Ti_3_C_2_T_x_, and Fe_1_‐N^S‐Fe_1_/Ti_3_C_2_T_x_ catalysts, initial screening tests were conducted in a customized air‐tight H‐type cell separated by a Nafion (117) membrane with 0.1 m Na_2_SO_4_ saturated N_2_ electrolyte solution at room temperature. Linear sweep voltammetry (LSV) measurements (**Figure** **4**a) were first acquired at applied potentials ranging from 0 to −1.4 V (with all potentials converted to a reversible hydrogen electrode, RHE) under both Ar and N_2_‐saturated Na_2_SO_4_ electrolytes to assess the catalytic performance of the Fe_1_‐N^S‐Ru_1_/Ti_3_C_2_T_x_. As shown in Figure x, the LSV curves exhibit a similar shape; however, there was a notable increase in current density below −0.1 V under N_2_ atmosphere, indicating the electrochemical response associated with eNRR. Subsequently, the chronoamperometric (CA) tests with continuous N_2_ bubbling for 2 h were performed to investigate the catalytic efficiency and optimal potential for the Fe_1_‐N^S‐Ru_1_/Ti_3_C_2_T_x_, Ru_1_‐N^S‐Ru_1_/Ti_3_C_2_T_x_, and Fe_1_‐N^S‐Fe_1_/Ti_3_C_2_T_x_ catalysts. As depicted in Figures S9–S11 (Supporting Information), each catalyst demonstrates a steady current density throughout a 2‐h electrolysis in Na_2_SO_4_ electrolyte. The corresponding NH_3_ yield rates and Faradaic efficiencies (FEs) at each applied potential were spectrophotometrically determined using the indophenol blue method (Figure S12, Supporting Information).^[^ ^44^ ^]^ From the results presented in Figure 4b,c, Fe_1_‐N^S‐Ru_1_/Ti_3_C_2_T_x_ distinctly outperformed the other two catalysts, achieving a significantly higher NH_3_ yield and FEs at otherwise identical conditions. Specifically, an NH_3_ yield rate of 32.8 µg h^−1^ mg^−1^_cat_ at −0.55 V and FE of 47.1% at −0.25 V were achieved, respectively. Notably, the NH_3_ yield rate is 3.2 times higher for Ru_1_‐N^S‐Ru_1_/Ti_3_C_2_T_x_ and 2.3 times higher for Fe_1_‐N^S‐Fe_1_/Ti_3_C_2_T_x_, while the FEs are ≈3.0 times higher for both Ru_1_‐N^S‐Ru_1_/Ti_3_C_2_T_x_ and Fe_1_‐N^S‐Fe_1_/Ti_3_C_2_T_x_. These results indicate that the formation of heteronuclear dual Ru‐Fe active sites with an asymmetric configuration is more favorable for the eNRR compared to their homonuclear dual Ru‐Ru or Fe‐Fe analogues with symmetric configurations.

Due to the high sensitivity of eNRR results to external factors that can introduce uncontrollable errors, a series of control experiments was conducted to validate the accuracy of the eNRR performance. Minimal or no NH_3_ was detected during eNRR measurements conducted in a blank electrolyte, N_2_‐saturated electrolyte at open circuit, Ar‐saturated electrolyte, or using carbon paper as the electrode. These observations strongly indicate that the produced NH_3_ originated exclusively from the electroreduction of N_2_ feeding gas in the electrolyte, catalyzed by Fe_1_‐N^S‐Ru_1_/Ti_3_C_2_T_x_. Furthermore, isotope‐labeling experiments using ^15^N_2_ gas under optimized conditions demonstrated eNRR activity comparable to that observed with N_2_ (Figure S13, Supporting Information), thereby excluding interference from other nitrogen sources. The CA measurements further confirm the stability of Fe_1_‐N^S‐Ru_1_/Ti_3_C_2_T_x_, showing minimal fluctuation in the current density over 50 h of electrolysis (Figure 4d). Additionally, the used Fe_1_‐N^S‐Ru_1_/Ti_3_C_2_T_x_ over 50 h of electroreduction exhibited negligible performance degradation in both NH_3_ yield rate and FEs, thereby reaffirming its high stability. Characterizations, including XPS and AC HAADF‐STEM, revealed no significant changes in the morphology and structure of the used Fe_1_‐N^S‐Ru_1_/Ti_3_C_2_T_x_ (Figures S14 and S15, Supporting Information). The endurance of Fe_1_‐N^S‐Ru_1_/Ti_3_C_2_T_x_ was evaluated over ten consecutive electrochemical cycles under optimized conditions. As shown in Figure 4e, while the NH_3_ yield rate and FEs slightly fluctuated between cycles, they remained stable throughout the eNRR process, indicating the exceptional chemical stability of Fe_1_‐N^S‐Ru_1_/Ti_3_C_2_T_x_.

**Figure 5:**
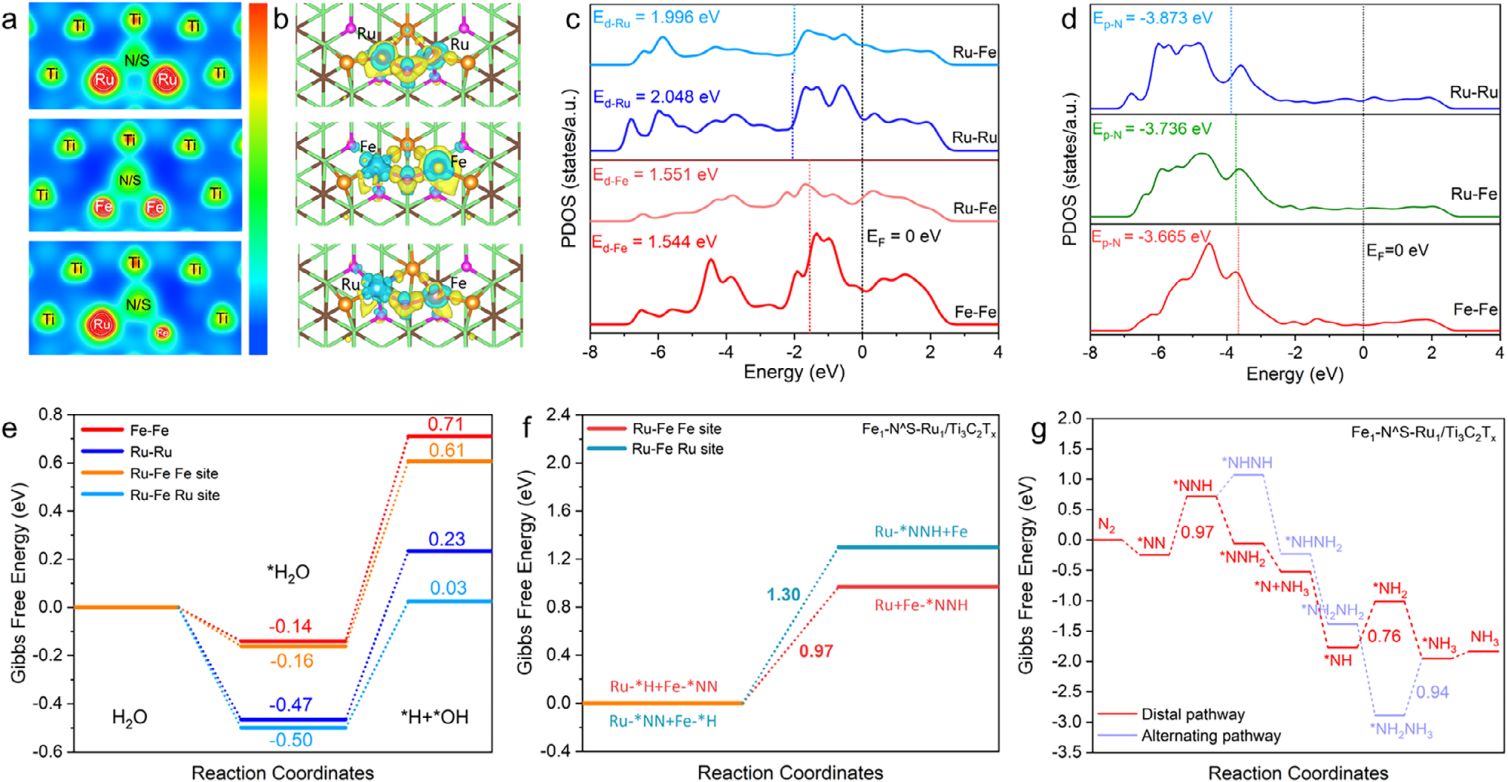
a) The electron localization function (ELF) plots of Isosurface of Ru_1_‐N^S‐Ru_1_/Ti_3_C_2_T_x_, Fe_1_‐N^S‐Fe_1_/Ti_3_C_2_T_x_, and Fe_1_‐N^S‐Ru_1_/Ti_3_C_2_T_x_. b) Charge density difference of Ru_1_‐N^S‐Ru_1_/Ti_3_C_2_T_x_, Fe_1_‐N^S‐Fe_1_/Ti_3_C_2_T_x_ and Fe_1_‐N^S‐Ru_1_/Ti_3_C_2_T_x_. c) PDOS of M_1_‐M_2_. d) PDOS of M_1_‐M_2_. e) The adsorption capability for H_2_O of different active sites. f) Gibbs free energy barrier for the hydrogenation of N_2_^*^ at metal active sites. g) Gibbs free energy diagrams for the distal and alternating pathway of NRR on Fe_1_‐N^S‐Ru_1_/Ti_3_C_2_T_x_.

DFT calculations were conducted to gain a deeper understanding of the origin of the exceptional catalytic activity on the Fe_1_‐N^S‐Ru_1_/Ti_3_C_2_T_x_. Three models were constructed to represent the catalysts based on the structural characterizations, that is, an asymmetric heteronuclear Ru‐Fe configuration for Ru_1_‐N^S‐Fe_1_/Ti_3_C_2_T_x_, and two symmetric configurations of homonuclear Ru‐Ru for Ru_1_‐N^S‐Ru_1_/Ti_3_C_2_T_x_ and Fe‐Fe for Fe_1_‐N^S‐Fe_1_/Ti_3_C_2_T_x_ (Figure S16, Supporting Information). The electron localization function (ELF) plots (**Figure** **5**a) provide an intuitive visualization of the changes in the electron distribution around Fe_1_‐N^S‐Ru_1_/Ti_3_C_2_T_x_. Compared to the symmetric homonuclear DACs Ru_1_‐N^S‐Ru_1_/Ti_3_C_2_T_x_ and Fe_1_‐N^S‐Fe_1_/Ti_3_C_2_T_x_, the heteronuclear DAC Fe_1_‐N^S‐Ru_1_/Ti_3_C_2_T_x_ exhibits a significantly higher electron density in the vicinity of the Ru atoms. The charge density difference plot of Fe_1_‐N^S‐Ru_1_/Ti_3_C_2_T_x_ reveals a clear electron transfer from the less electronegative Fe atom to the Ru through the bridged Fe─Ru bond interaction (Figures 5b; Table S3, Supporting Information). The projected density of states (PDOS) (Figure 5c,d) demonstrates that the formation of asymmetric heteronuclear dual Fe‐Ru sites results in an upward shift of the d‐band center of Ru from −2.048 eV in the symmetric homonuclear Ru_1_‐N^S‐Ru_1_/Ti_3_C_2_T_x_ to −1.996 eV in Fe_1_‐N^S‐Ru_1_/Ti_3_C_2_T_x_. Concurrently, the d‐band center of Fe exhibits a slightly downward shift from −1.544 eV in the symmetric homonuclear Fe_1_‐N^S‐Fe_1_/Ti_3_C_2_T_x_ to −1.551 eV in Fe_1_‐N^S‐Ru_1_/Ti_3_C_2_T_x_. These opposite shifts in d‐band center confirm the electron transfer from Fe to Ru sites (Figure S17, Supporting Information). These computational findings are consistent with the results obtained from XPS and XANES analysis, verifying the electron transfer and redistribution between Ru and Fe sites in the asymmetric heteronuclear DAC Fe_1_‐N^S‐Ru_1_/Ti_3_C_2_T_x_.

Further calculations were conducted to investigate the adsorption and activation capabilities of N_2_ and H_2_O molecules on the catalyst's surface, which are two key factors influencing eNRR performance.^[^ ^16^ ^]^ The heteronuclear DAC Fe_1_‐N^S‐Ru_1_/Ti_3_C_2_T_x_ with asymmetric dual Ru‐Fe sites demonstrates a stronger ability for N_2_ adsorption with calculated adsorption energies (Table S4, Supporting Information), when compared with the homonuclear DAC Ru_1_‐N^S‐Ru_1_/Ti_3_C_2_T_x_ and Fe_1_‐N^S‐Fe_1_/Ti_3_C_2_T_x_ with symmetric dual sites (−0.13 and −0.16 eV). Notably, the N_2_ molecules could adsorb on either the Ru sites or the Fe sites of the heteronuclear Fe_1_‐N^S‐Ru_1_/Ti_3_C_2_T_x_, however, calculations reveal that N_2_ adsorption on the Fe site is significantly stronger than that on the Ru site (−0.55 vs −0.33 eV). This indicates a preferential chemisorption and activation of N_2_ molecules at the Fe sites in the heterogeneous DAC Fe_1_‐N^S‐Ru_1_/Ti_3_C_2_T_x_. Furthermore, the N_2_ adsorption energy is relatively higher on the homonuclear DAC Fe_1_‐N^S‐Fe_1_/Ti_3_C_2_T_x_ (−0.16 eV) than on Ru_1_‐N^S‐Ru_1_/Ti_3_C_2_T_x_ (−0.13 eV), providing further evidence for a preferable N_2_ adsorption site on the Fe atom due to its intrinsic affinity to N_2_ molecules.^[^ ^45^, ^46^ ^]^ The calculation results are corroborated by N_2_‐temperature programmed desorption (N_2_‐TPD) (Figure S18, Supporting Information), where Fe_1_‐N^S‐Ru_1_/Ti_3_C_2_T_x_ exhibits significantly higher peak intensity and area for N_2_ desorption, following the trend of Fe_1_‐N^S‐Ru_1_/Ti_3_C_2_T_x_ > Fe_1_‐N^S‐Fe_1_/Ti_3_C_2_T_x_ > Ru_1_‐N^S‐Ru_1_/Ti_3_C_2_T_x_, aligning well with the calculated result. In parallel, calculation (Figure 5f) shows that the heteronuclear DAC Fe_1_‐N^S‐Ru_1_/Ti_3_C_2_T_x_ with asymmetric dual Ru‐Fe sites also enhances the adsorption capability for H_2_O, with an energy of −0.50 eV compared to −0.47 eV for the homonuclear DAC Ru_1_‐N^S‐Ru_1_/Ti_3_C_2_T_x_ and −0.16 eV for Fe_1_‐N^S‐Fe_1_/Ti_3_C_2_T_x_. Moreover, we calculated the activation energy for H_2_O dissociation to produce H^*^ species needed for N_2_ hydrogenation across the three catalysts. As shown in Figure 5e, an energy barrier of 0.03 eV is required to break the H─OH bond for dissociation on the Ru sites in the heteronuclear Fe_1_‐N^S‐Ru_1_/Ti_3_C_2_T_x_, which is significantly lower than on the Fe sites (0.61 eV). This suggests a preferable H_2_O activation to generate H protons on the Ru sites in Fe_1_‐N^S‐Ru_1_/Ti_3_C_2_T_x_. Notably, the generation of H^*^ species requires overcoming an energy barrier of 0.71 eV on the homonuclear DAC Fe_1_‐N^S‐Fe_1_/Ti_3_C_2_T_x_ with symmetric dual Fe‐Fe sites, indicating that H_2_O activation on the Fe sites is more challenging. Additionally, the formation of asymmetric heteronuclear Fe_1_‐N^S‐Ru_1_/Ti_3_C_2_T_x_ also significantly reduces the dissociation energy of H_2_O compared to the symmetric homonuclear Ru_1_‐N^S‐Ru_1_/Ti_3_C_2_T_x_ (0.03 vs 0.23 eV), thereby facilitating the generation of H^*^ species, beneficial for the enhancement of eNRR. In addition, we calculated the kinetic energy barriers for the proton transfer to the activated ^*^N_2_ on the catalyst surface to form ^*^NNH, which is widely recognized as the potential‐determining step (PDS) for eNRR.^[^ ^47^ ^]^ As shown in Figure 5f, the transfer of ^*^H from the Ru site to the activated ^*^N_2_ adsorbed on the Fe site requires overcoming an energy barrier of 0.97 eV, considerably lower than that of the reverse ^*^H transfer from the Fe site to ^*^N_2_ adsorbed on the Ru site (1.30 eV). This further confirms that N_2_ molecules preferentially adsorb on the Fe sites for activation, while the Ru sites facilitate H_2_O dissociation to produce H^*^, thereby boosting the eNRR in a cooperative and synergistic manner due to the spatial proximity of dual Fe‐Ru sites in the asymmetric heteronuclear DAC Fe_1_‐N^S‐Ru_1_/Ti_3_C_2_T_x_. In situ Raman spectroscopy also validates these findings (Figure S19, Supporting Information), in which a characteristic vibration at ≈150 cm^−1^, corresponding to the generated Fe‐N^*^ intermediate species, was observed.^[^ ^48^, ^49^ ^]^ Additionally, we calculated the energy barriers of the PDS for the presence of isolated mononuclear Ru or Fe sites. As shown in Figure S20 (Supporting Information), a significantly higher energy barrier must be overcome in these cases, thereby effectively ruling out their contribution to eNRR efficiency.

Lastly, to investigate the mechanism of the eNRR process on the catalysts Fe_1_‐N^S‐Ru_1_/Ti_3_C_2_T_x_, Ru_1_‐N^S‐Ru_1_/Ti_3_C_2_T_x_, and Fe_1_‐N^S‐Fe_1_/Ti_3_C_2_T_x_, the Gibbs free energies of each elementary step were calculated. Both the distal pathway and the alternating pathway, which are widely accepted mechanisms for eNRR^[^ ^14^ ^]^ were considered. As shown in Figure 5g and Figures S21 and S22 (Supporting Information) our calculations reveal that the reaction primarily proceeds through the distal pathway, as it exhibits an overall lower energy barrier compared to the alternating pathway. The initial step of proton‐coupled electron transfer that generate ^*^NNH from the adsorbed ^*^N_2_ serves as the potential‐determining step (PDS), requiring the largest uphill energy input among all tested catalysts across each elementary reaction step. The asymmetric heteronuclear DAC Fe_1_‐N^S‐Ru_1_/Ti_3_C_2_T_x_ demonstrates the lowest reaction energy barrier of 0.97 eV for the PDS, in comparison to 1.24 eV for the symmetric homonuclear Fe_1_‐N^S‐Fe_1_/Ti_3_C_2_T_x_ and 1.37 eV for Ru_1_‐N^S‐Ru_1_/Ti_3_C_2_T_x_. This calculated trend in reaction energy barriers for the PDS coincides well with the experimentally observed eNNR activities of the catalysts. In a word, the formation of asymmetric heteronuclear DAC Fe_1_‐N^S‐Ru_1_/Ti_3_C_2_T_x_ substantially reduces the energy barrier in the PDS, thereby promoting the overall kinetics of the eNRR.

## 3 Conclusion

In summary, we developed a heteronuclear dual Ru‐Fe site with asymmetric configuration anchored on N,S‐codoped Ti_3_C_2_T_x_. Both experimental results and DFT theoretical calculations revealed that the asymmetric coordination of RuFe modulates the electronic distribution and spatial arrangement between active sites, effectively adsorbing and activating ^*^N_2_ while reducing the Gibbs free energy barriers for the subsequent hydrogenation of N intermediates. Fe_1_‐N^S‐Ru_1_/Ti_3_C_2_T_x_ exhibited an NH_3_ production rate of 32.8 µg h^−1^ mg^−1^_cat_ at −0.55 V versus RHE and FE of 47.1% at −0.25 V versus RHE. This study confirms that the NS‐bridged dual single‐atom strategy not only preserves the intrinsic catalytic properties of metal sites but also promotes synergistic interactions between neighboring atoms, providing valuable guidance for the synthesis of future heterogeneous dual single‐atom catalysts. The investigation of the synergistic effects in NRR contributes to advancing the development of NRR catalysis.

## 4 Experimental Section

### Materials

Ti_3_AlC_2_ powder was purchased from 11 Technology Co., Ltd (China). Unless otherwise noted, all reagents were purchased commercially from Sigma‐Aldrich or Aladdin and used as received without further purification.

### Preparation of 2D Layered Ti_3_C_2_T_x_ MXene Nanosheets

First, 2 g of LiF was dissolved in 40 mL of 9 m HCl solution and stirred at 350 rpm for 30 min. Then 2 g of Ti_3_AlC_2_ powder was slowly added to the above solution and stirred at 35 °C for 24 h. The resultant solution was washed several times using deionized water until the pH of the supernatant reached 5. The sediment was collected and mixed with 50 mL of deionized water, which was further sonicated for 1 h under an Ar atmosphere, followed by centrifugation at 3500 rpm for 1 h to obtain the Ti_3_C_2_T_x_ MXene suspension. Ti_3_C_2_T_x_ MXene powder was prepared by freeze‐drying of Ti_3_C_2_T_x_ MXene suspension.

### Preparation of Fe_1_‐N^S‐Ru_1_/Ti_3_C_2_T_x_

A solution of 0.5 mL of 15 mm FeCl_3_ and 0.5 mL of 15 mm RuCl_3_ was thoroughly mixed with 100 mg of thiourea dissolved in a solvent, followed by the addition of a monolayer of Ti_3_C_2_T_x_ aqueous dispersion to form a homogeneous mixture under vigorous stirring. The resulting mixture was freeze‐dried to obtain the catalyst precursor. The precursor was then subjected to pyrolysis under an Ar atmosphere at 500 °C for 2 h (ramp rate, 5 °C min^−1^), and the N and S elements generated from the decomposition of thiourea were doped into the Ti_3_C_2_T_x_ skeleton in situ, while anchoring Fe and Ru species to form bimetallic active sites. For comparative studies, symmetric binuclear Ru–Ru and Fe–Fe paired‐site catalysts (denoted as Ru_1_‐N^S‐Ru_1_/Ti_3_C_2_T_x_ vs Fe_1‐_N^S‐Fe_1_/Ti_3_C_2_T_x_) were prepared using the same methodology (only a single metal precursor solution was introduced), respectively.

### Characterization

The X‐ray diffraction (XRD) patterns were obtained on a Bruker D8 Advance diffractometer equipped with Cu Kα radiation (λ = 1.5147 Å). A transmission electron microscope (TEM) was used to record using a Hitachi H‐7600. High‐resolution TEM (HRTEM) and scanning transmission electron microscope (STEM) images were recorded using FEI Tecnai G2 F30. The X‐ray photoelectron spectroscopy (XPS) was conducted on an ESCALAB 250Xi (Thermo Scientific, UK) instrument using an Al Kα line source. A monochromatic Al Kα X‐ray source (hν = 1486.6 eV) was used. The source was operated at 15 kV and 10 mA, providing a power of 150 W. To mitigate charging effects on the insulating sample, a combined low‐energy electron and argon ion flood gun was used. The analysis area was ≈500 µm. Survey spectra were collected with a pass energy of 100 eV and a step size of 1.0 eV to identify all elements present. All spectra were charge‐corrected to compensate for any residual surface charging. The adventitious carbon C 1s peak was set to a binding energy of 284.8 eV. Data processing and peak fitting were performed using Avantage software. A Smart background was subtracted from all high‐resolution spectra prior to peak fitting. The choice of background was consistent across all comparable samples. Peak fitting was conducted using a combination of Gaussian‐Lorentzian line shapes (GL), specifically a [e.g., 70% Gaussian / 30% Lorentzian, or Voigt] profile. This product form (GL%) is standard for replicating the natural line shape of XPS peaks. The elemental analysis was recorded using a Vario El elemental analyzer. The Raman spectra were measured on a Horiba Jobin Yvon LabRAM HR800. The absorbance data of the electrolyte were obtained by a UV–vis spectrophotometer (Lambda 25, PerkinElmer). X‐ray absorption spectroscopy (XAS) measurements were carried out using a Si (311) double‐crystal monochromator (NW10A, PF‐AR) at room temperature at the Institute of Materials Structure Sciences of High Energy Accelerator Research Organization (KEK). The monochromator energy was calibrated using a Fe and Ru foil. The XAFS data were analyzed using IFEFFIT. The XAFS raw data were background subtracted, normalized, and Fourier transformed by standard procedure within the ATHENA program. The acquired EXAFS data were processed according to the standard procedures using the ATHENA module implemented in the IFEFFIT software packages. The *k^3^*‐weighted EXAFS spectra were obtained by subtracting the post‐edge background from the overall absorption and then normalizing with respect to the edge‐jump step. Subsequently, *k^3^*‐weighted χ(k) data of Ru and Ke K‐edge were Fourier transformed to real (R) space using a Hanning window (d_k_ = 1.0 Å^−1^) to separate the EXAFS contributions from different coordination shells. To obtain the quantitative structural parameters around central atoms, least‐squares curve parameter fitting was performed using the ARTEMIS module of the IFEFFIT software packages. Data processing and fitting were performed using the Athena and Artemis software packages. The fitting in R‐space was conducted over a range of 1–3 Å, with the k‐space fitting range set from 3.0 to 14.95 Å. The Ru/Fe─N/S bonds derived from the CIF files were used as fitting paths to model the R‐space EXAFS spectra for Ru/Fe‐N/S coordination. By adjusting parameters including different scattering paths and coordination numbers, the final R‐factor was optimized to below 0.02 (detailed parameters are provided in Table S2, Supporting Information). The CIF files used in this analysis include: Ru_2_C_6_O_8_‐7106715.cif; Ru_3_C_12_O_12_‐1502714.cif; Fe_3_N‐1011217.cif; FeNS‐7226300.cif; FeS_2_‐1010760.cif et al. Electrochemical NRR measurements were performed over an electrochemical working station (CHI 660E, Shanghai CH Instruments Co., China). N_2_ temperature programmed desorption (N_2_‐TPD) was measured on AMI‐300 automatic chemisorption analyser, test temperature range of 50–800 °C with a ramp rate of 10 °C min^−1^. The in‐situ Raman test utilized the DXR3xi Raman imaging microscope to determine the Raman spectra of the catalysts in the in situ NRR process under different operating voltage conditions (0−1.0 V vs RHE).

### Electrocatalytic NRR Measurements

All electrochemical NRR measurements were performed using a CHI‐660E electrochemical workstation equipped with a three‐electrode system. The double‐chamber electrolytic cell is divided into two parts by a Nafion‐117 membrane. Each chamber is filled with 50 mL of 0.1 m Na_2_SO_4_ solution as electrolyte, and platinum foil and Ag/AgCl electrodes are used as counter electrode and reference electrode, respectively. Before the NRR process test, the electrolyte was purged using continuous N_2_ gas flow (50 mL min^−1^) for 30 min. All potentials in the experiment are converted to the reversible hydrogen electrode (RHE) by Eq. (1):

(1) $$E\;\left(vs.\;RHE\right)=E\left(vs.Ag/AgCl\right)+0.059\times pH+0.197V$$

### Determination of Ammonia and Hydrazine

The concentration of produced NH_3_ and hydrazine in the electrolyte was determined by the indophenol blue method and the Watt and Chrisp method, respectively. The NH_3_ yield ($r_{NH_{3}}$) and FE were calculated from Eq. (2):

(2) $$\begin{array}{c} r_{NH_{3}}=\frac{c_{NH_{3}}\times V}{t\times m}\\ FE=3F\times \frac{c_{NH_{3}\times V}}{Q} \end{array}$$

where $c_{NH_{3}}$ (µg mL^−1^) is the measured NH_3_ concentration; V (mL) is the volume of electrolyte (typically 50 mL); t (h) is the electrochemical reaction time (typically 2 h); m (mg) is the mass of supported catalyst; F stands for Faraday's constant, and its value is 96 485 C mol^−1^; Q is the total charge consumed in the electrolysis process.

### 15N_2_ Isotope Labeling Experiments

The ^15^N isotopic experiment was conducted using the ^15^N_2_ to certify the N origin of ammonia. To exclude the dissolved N_2_ in the electrolyte, the electrolyte was pre‐treated. Then, 50 mL pre‐treated electrolyte was purged with Ar (99.999%) gas for 30 min. Before the experiment,^15^N_2_ was purged into the electrolyte at very low flow rates to remove any possible air in the equipment. After 2 h electrochemical process, the electrolyte was concentrated to 3 mL through a decompress distillation process at 60 °C, and then mixed with 550 µL d6‐DMSO. The mixed solutions were analysed through the ^1^H NMR measurement (600 MHz) to detect the NH_3_ product.

## Conflict of Interest

The authors declare no conflict of interest.

## Supporting information



Supporting Information

## Data Availability

The data that support the findings of this study are available in the supplementary material of this article.
