# Supplementary material for: Synergistic Electrocatalytic N2 Reduction over Asymmetric Heteronuclear Dual Ru‐Fe Sites
Source: Adv Sci (Weinh). 2025 Oct 14;12(48):e12218. doi: 10.1002/advs.202512218 (PMC12752592; doi:10.1002/advs.202512218)
Supplement: Supplementary file 1 — Supporting Information [file ADVS-12-e12218-s001.docx]

**Supplementary Information**

**Synergistic Electrocatalytic N_2_ Reduction Over Asymmetric Heteronuclear Dual Ru-Fe Sites**

*Zihao Yang*, Chao Feng, Yifan Liu, Yong Yang**

Z. Yang

College of Biological and Chemical Engineering, Qilu Institute of Technology, Jinan 250200, China.

E-mail: yangzh@qlit.edu.cn

Z. Yang, Y. Liu, Y. Yang

State Key Laboratory of Photoelectric Conversion and Utilization of Solar Energy, Qingdao Institute of Bioenergy and Bioprocess Technology, Chinese Academy of Sciences, Qingdao 266101, China

Y. Liu, Y. Yang

University of Chinese Academy of Sciences, Beijing 100049, China

C. Feng

College of Chemical and Biological Engineering, Shandong University of Science and Technology, Qingdao, Shandong 266590, China

Y. Yang

Shandong Energy Institute, Qingdao 266101, China

E-mail: yangyong@qibebt.ac.cn

**Models and Computational Methods**

All calculations were performed using the density functional theory (DFT) technique using the Vienna ab initio simulation package (VASP). Spin-polarized calculations were performed using the generalized gradient approximation (GGA) combined with the Perdew–Burke–Ernzerhof (PBE) method to determine the exchange and correlation energies. The projector-augmented wave (PAW) method was used to represent the core–valence electron interactions. The typical plane-wave cutoff energy was 400 eV for basis-set expansion. For geometry optimization calculations, forces were converged below 0.03 eV/Å. The SCF convergence energy was 1 × 10^–4^ eV. A 1 × 1 × 1 k-point mesh was used to perform all the calculations.

Three-layer slab model surfaces of Ti_3_C_2_ (MXene) substitutions were built to calculate the adsorption energies and Gibbs free energies. Three p (3 × 3) unit cell expansions were used to model the surface of Ti_3_C_2_ (XMane) with Ru-Ru, Ru-Fe, Fe-Fe dual atom doping. A vacuum of 15 Å was used to simulate the surface under periodic boundary conditions.

The adsorption energy (E_ads_) of the H_2_O and the other molecule on the surface was calculated as follows (eq 1):

$$E_{\mathrm{ads}}=E_{adsorbate+surface}-E_{\mathrm{surface}}-E_{\mathrm{gas}}, (1)$$

where E_surface_ is the clean surface relaxation energy of the surface slab, E_gas_ is the energy of a free gas molecule under conditions of vacuum, and E_adsorbate+surface_ is the energy of the composite system.

According to the definition of ΔG ^[1-3]^,

$\Delta G=\Delta E+\Delta E_{ZPE}-T\Delta S+\Delta G_{U}+\Delta G_{pH}+\Delta G_{field}$ (11)

ΔE is directly calculated by DFT.

ΔE_ZPE_ is obtained by calculating the vibration frequency of the intermediate (the ZPE corrects for the vibration of adsorbate at 0 K). Due to the conservation of elements before and after each calculation step, in the calculation, ΔE_ZPE_ can be approximated as 0.

Because of the temperature we defined as 0 K, TΔS = 0 ^[4]^.

ΔG_pH_ is the contribution of H^+^ concentration change to the Gibbs free energy. Due to the absence of pH changes in the gas-solid reaction, it can be ignored as 0 ^[5]^.

ΔG_U_ is the contribution of free energy in electrode potential. Electrocatalysis is not involved in this experiment, so it is ignored.

The correction term for electric double layer free energy, known as ΔG_field_, has negligible effect on overall free energy and can be ignored ^[3]^.

As the calculations are performed at 0 K at a fixed cell volume, the differences in the Gibbs free energy should equal the differences in the total energy. By this definition, a negative value of E_ads_ corresponds to exothermic and spontaneous adsorption processes.

The barrier energy, ΔE, for a difference between transition state energy difference and standard product formation enthalpy is defined as follows. The energy barrier calculation of H_2_O to H-OH on Ru-Ru as an example (eq 2):

$$\Delta E=\left( E_{H-OH-Ru-Ru}-E_{stab-Ru-Ru} \right)-(E_{H2O-Ru-Ru}-E_{stab-Ru-Ru}) (2)$$

where E_H2O-Ru-Ru_ and E_H-OH-Ru-Ru_ are the energies of the H_2_O and H-OH adsorbed on Ru-Ru, E_stab-Ru-Ru_ is the energy of the Ru-Ru structure surface slab. As the calculations are performed a t =0 K at a fixed cell volume, the differences in the Gibbs free energy should equal the differences in the total energy. By this definition, the lower the ΔE is, the easier it is to react.

**Supplementary Figures and Tables**

**
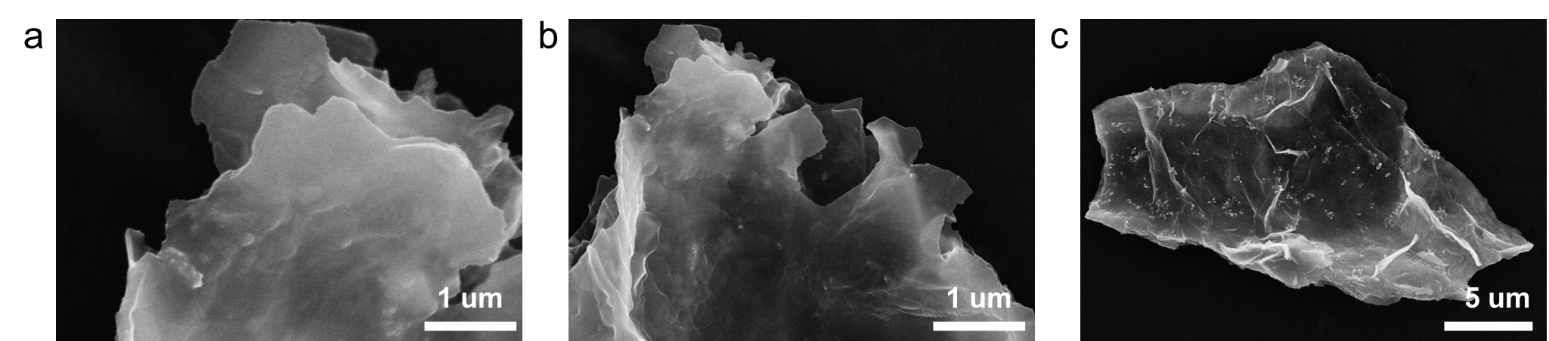
**

**Figure S1.** SEM images of Ru_1_-N^S-Ru_1_/Ti_3_C_2_T_x_, Fe_1_-N^S-Ru_1_/Ti_3_C_2_T_x_ and Fe_1_-N^S-Fe_1_/Ti_3_C_2_T_x_.


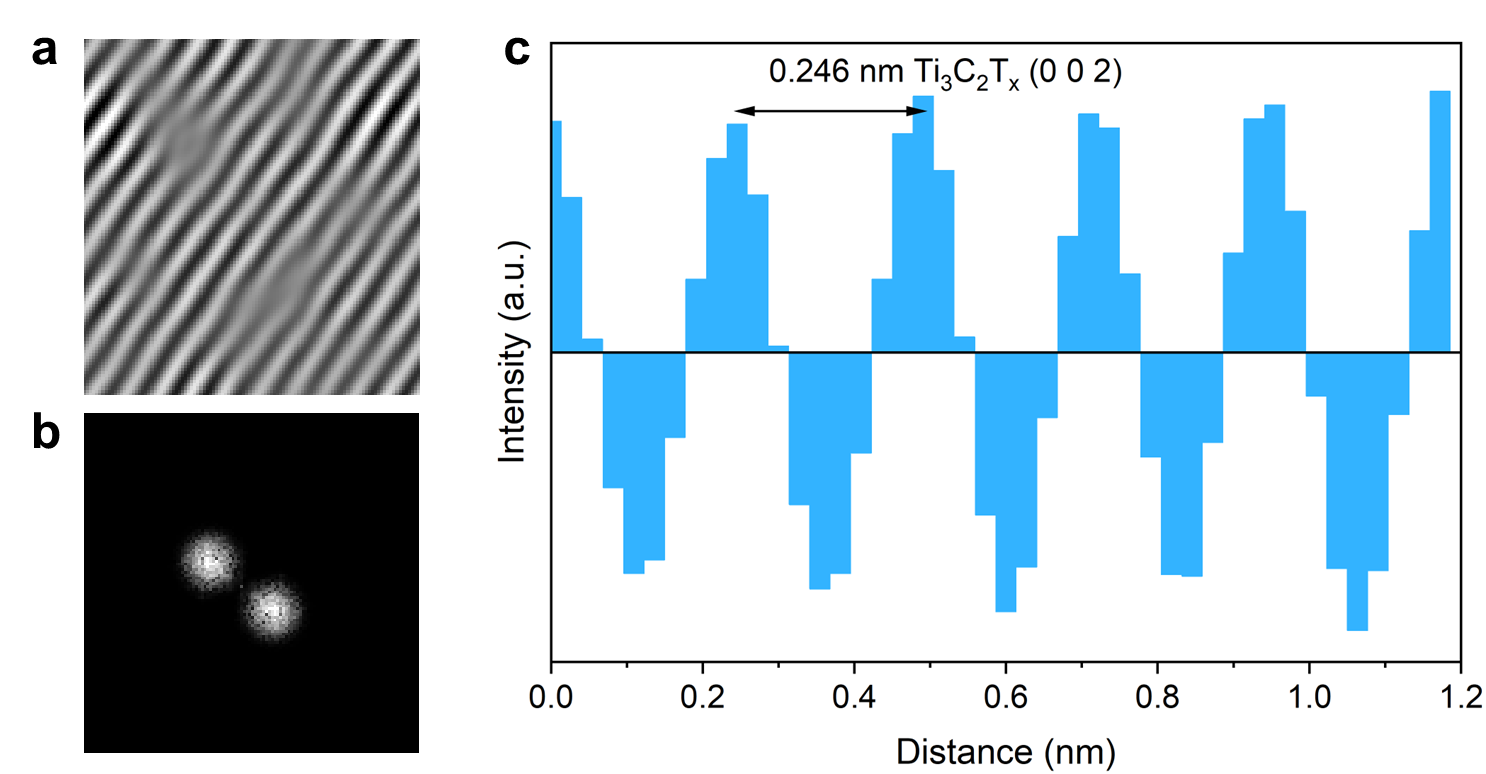


**Figure S2.** HRTEM images (a, b) and lattice spacing (c) for Fe_1_-N^S-Ru_1_/Ti_3_C_2_T_x_.


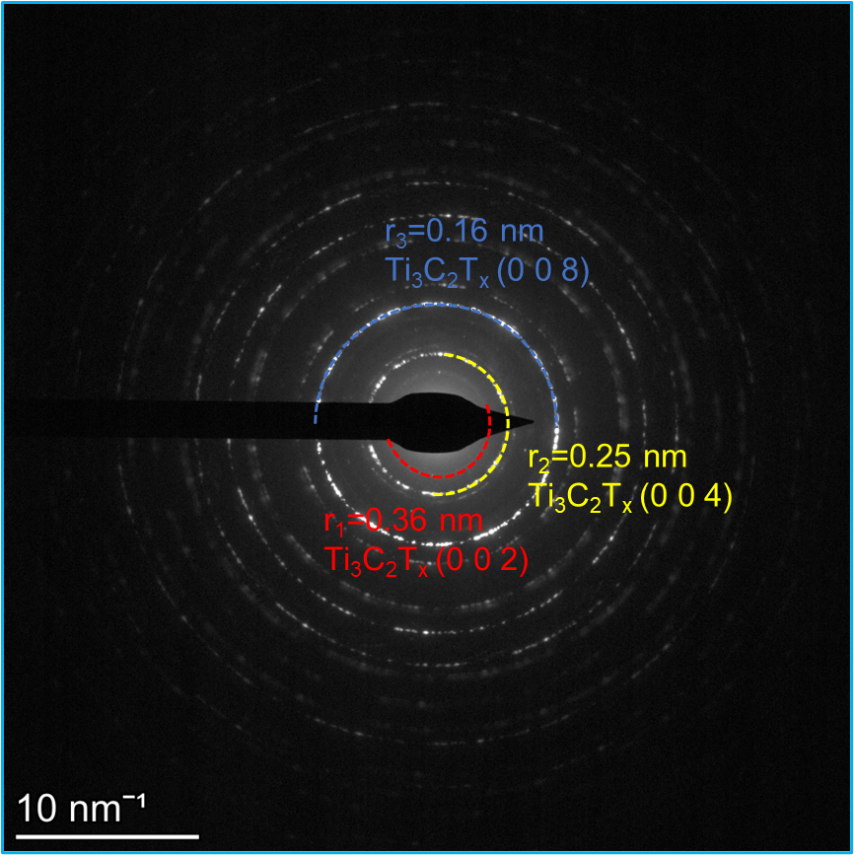


**Figure S3.** SAED images of Ru-Ti_3_C_2_T_x_-TEPS-2.


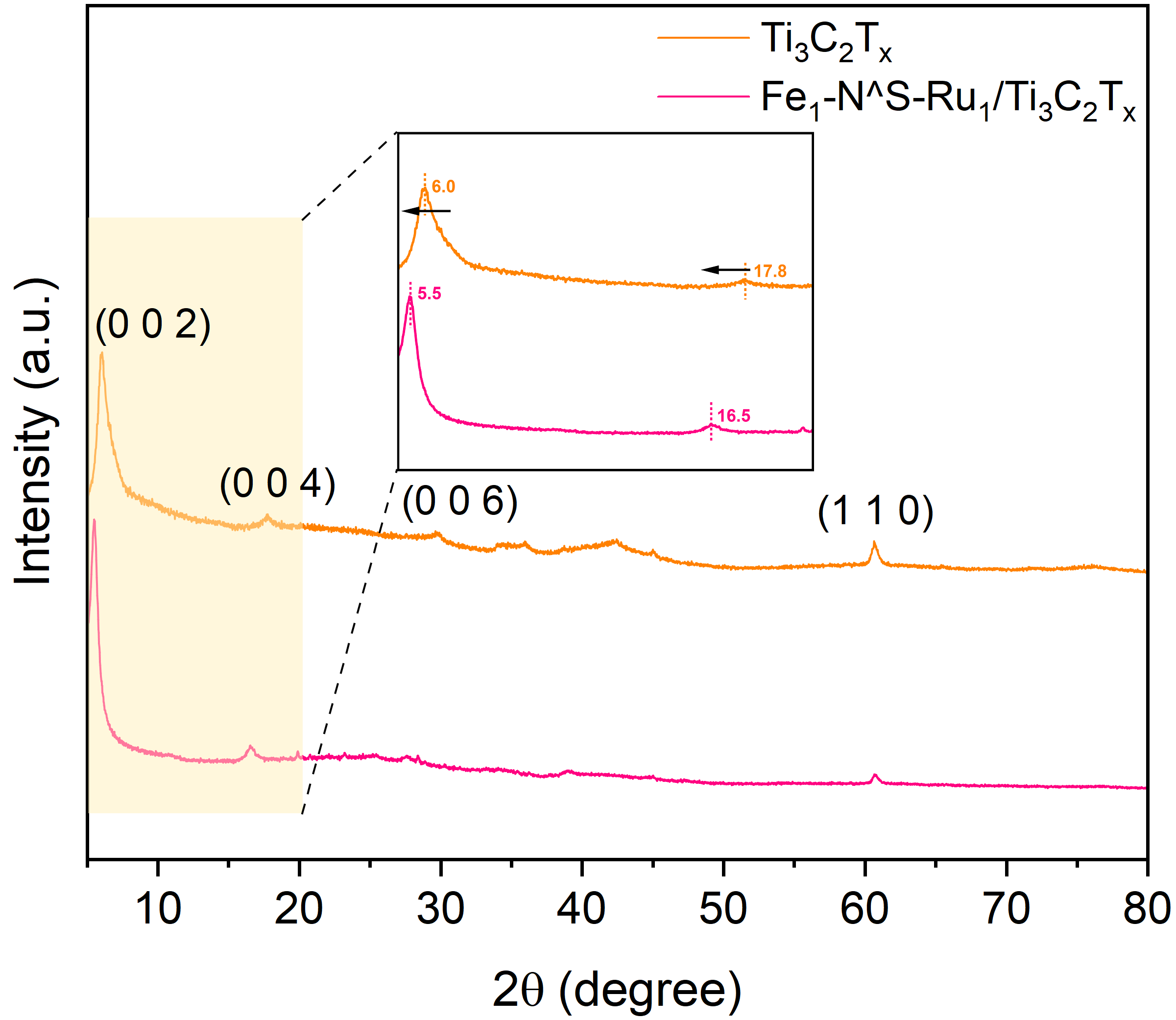


**Figure S4.** XRD pattern of Fe_1_-N^S-Ru_1_/Ti_3_C_2_T_x_ and Ti_3_C_2_T_x_.


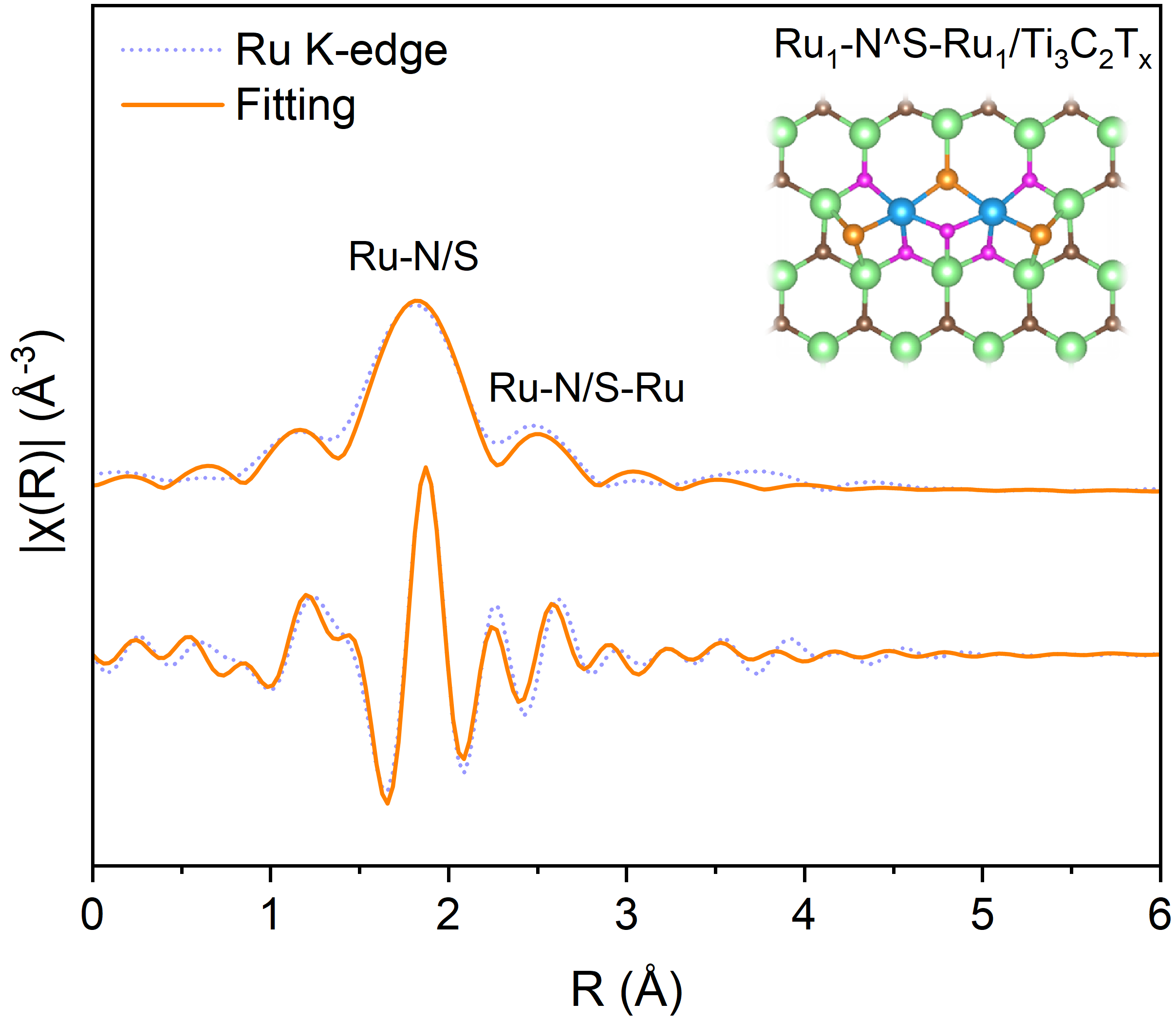


**Figure S5.** R-space curve-fitting of Ru_1_-N^S-Ru_1_/Ti_3_C_2_T_x_. The inset structure represents the atomic coordination fitting model.


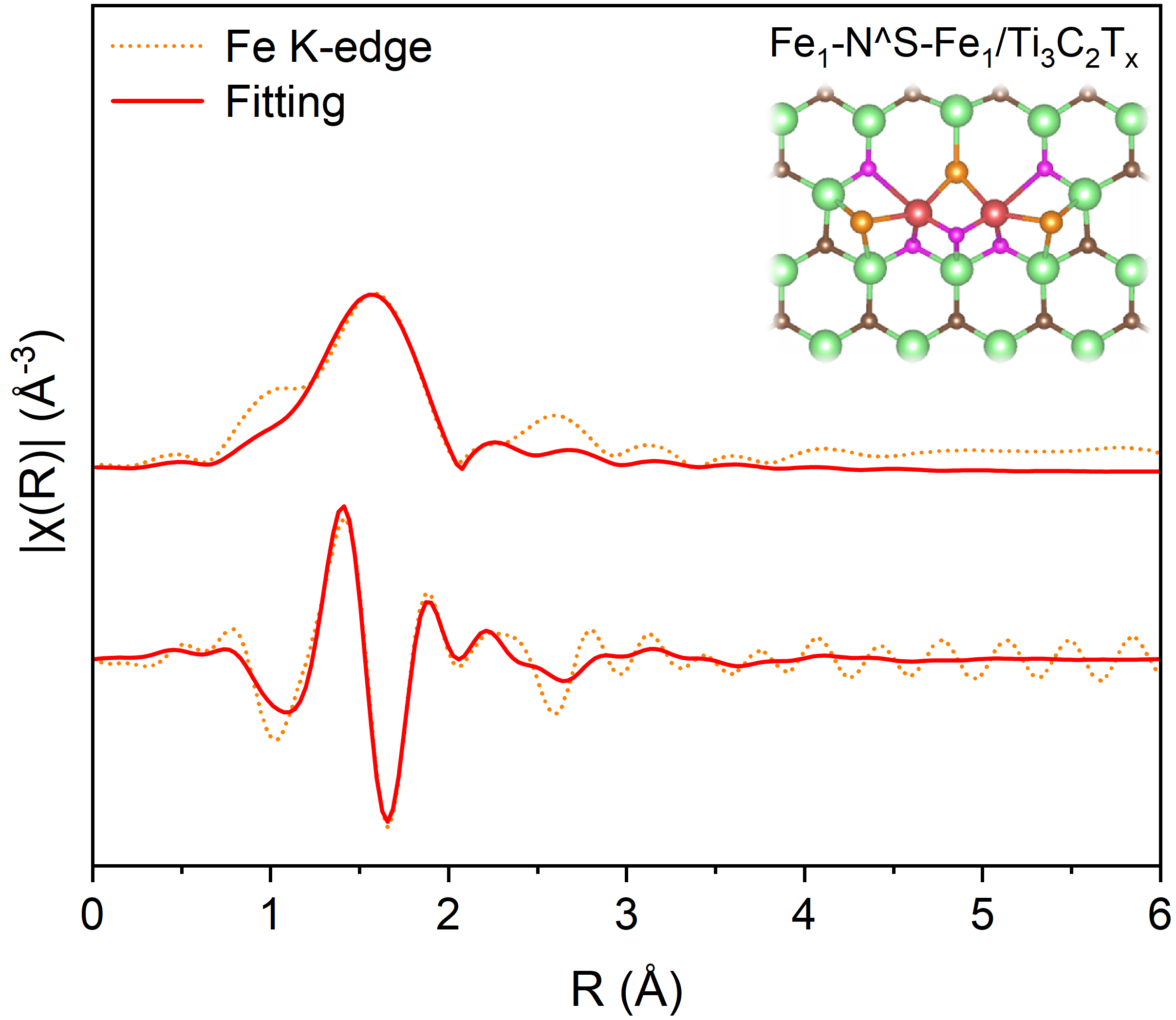


**Figure S6.** R-space curve-fitting of Fe_1_-N^S-Fe_1_/Ti_3_C_2_T_x_. The inset structure represents the atomic coordination fitting model.

**
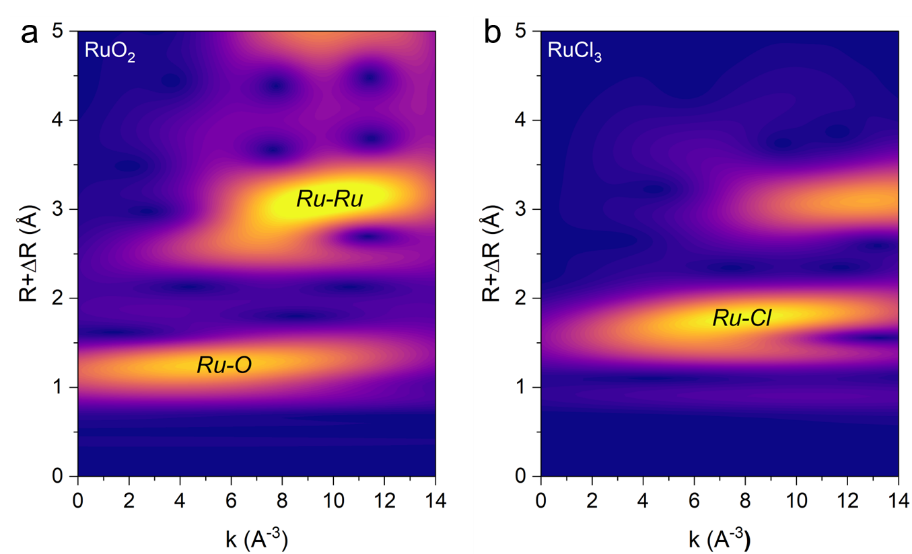
**

**Figure S7.** The WT-EXAFS spectra of a) RuO_2_ and b) RuCl_3_.


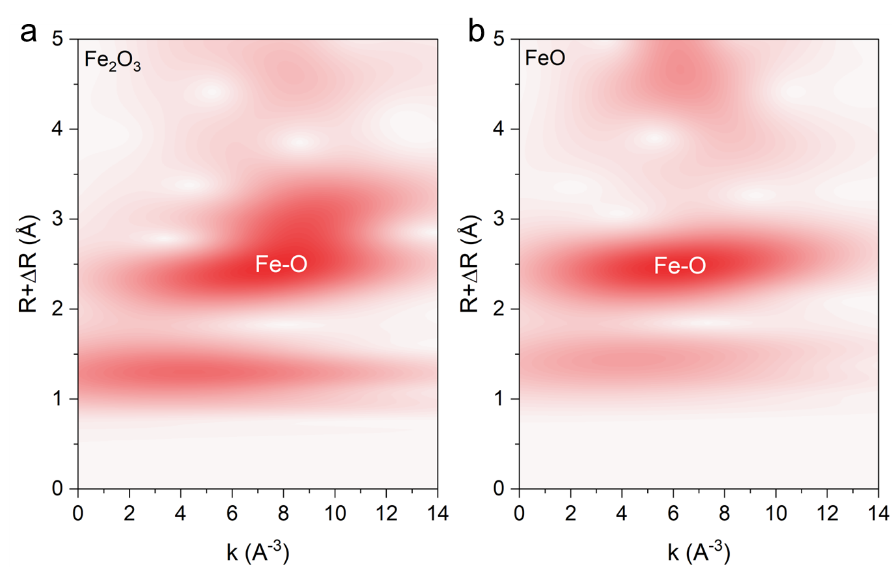


**Figure S8.** The WT-EXAFS spectra of a) Fe_2_O_3_ and b) FeO.

**
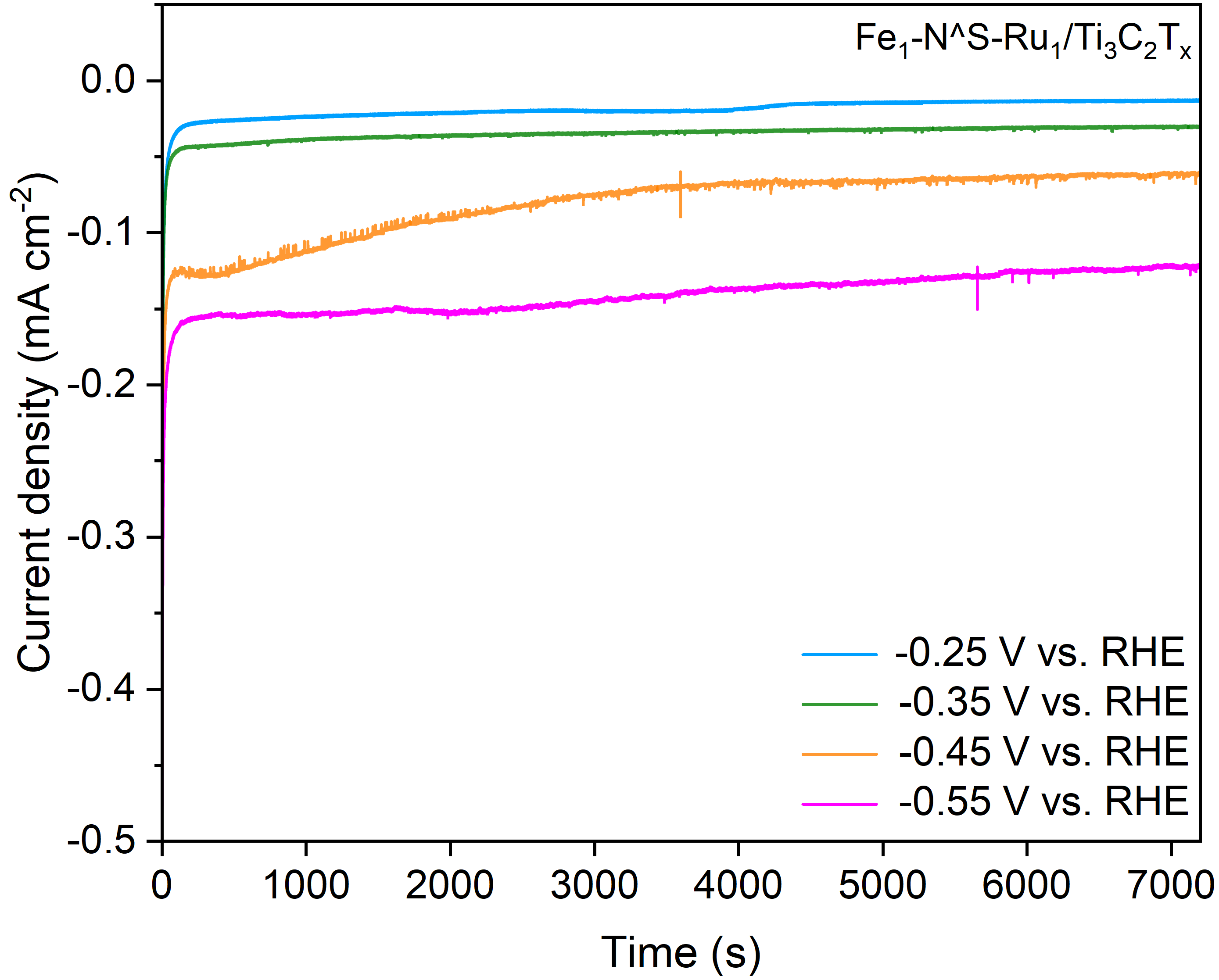
**

**Figure S9.** i-t curves of Fe_1_-N^S-Ru_1_/Ti_3_C_2_T_x_ catalysts under different potentials in 0.1 M Na_2_SO_4_ solution.


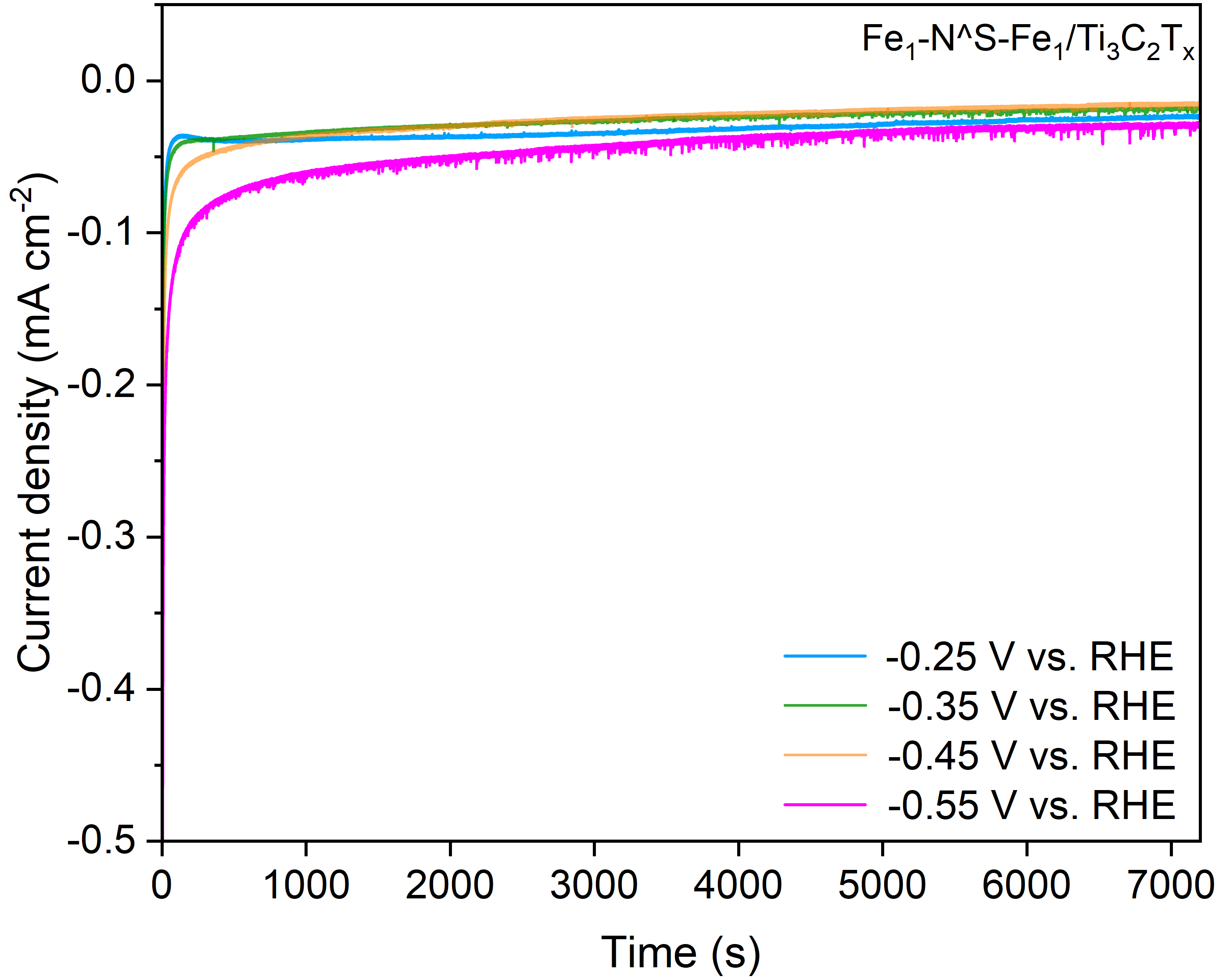


**Figure S10.** i-t curves of Fe_1_-N^S-Fe_1_/Ti_3_C_2_T_x_ catalysts under different potentials in 0.1 M Na_2_SO_4_ solution.

**
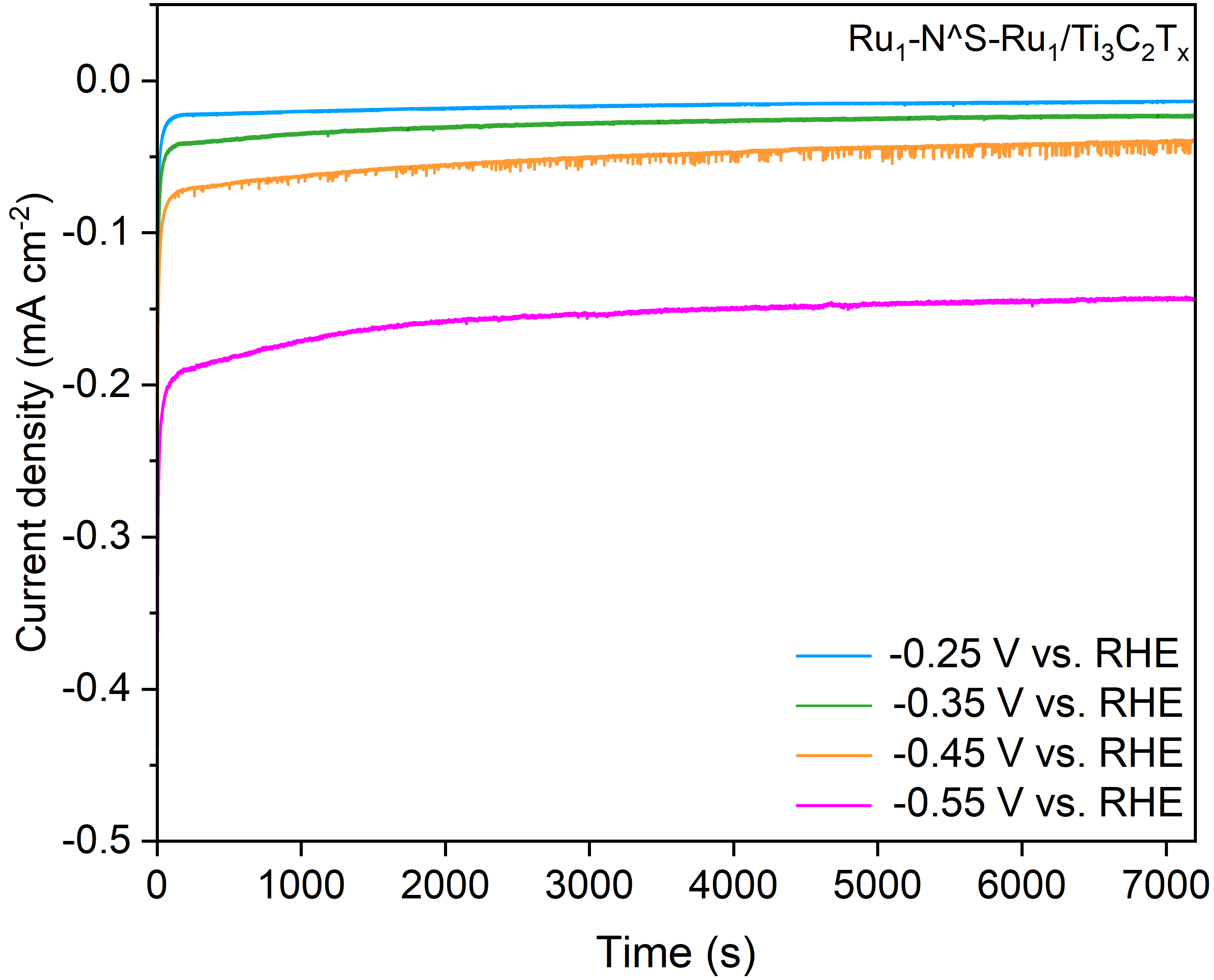
**

**Figure S11.** i-t curves of Ru_1_-N^S-Ru_1_/Ti_3_C_2_T_x_ catalysts under different potentials in 0.1 M Na_2_SO_4_ solution.


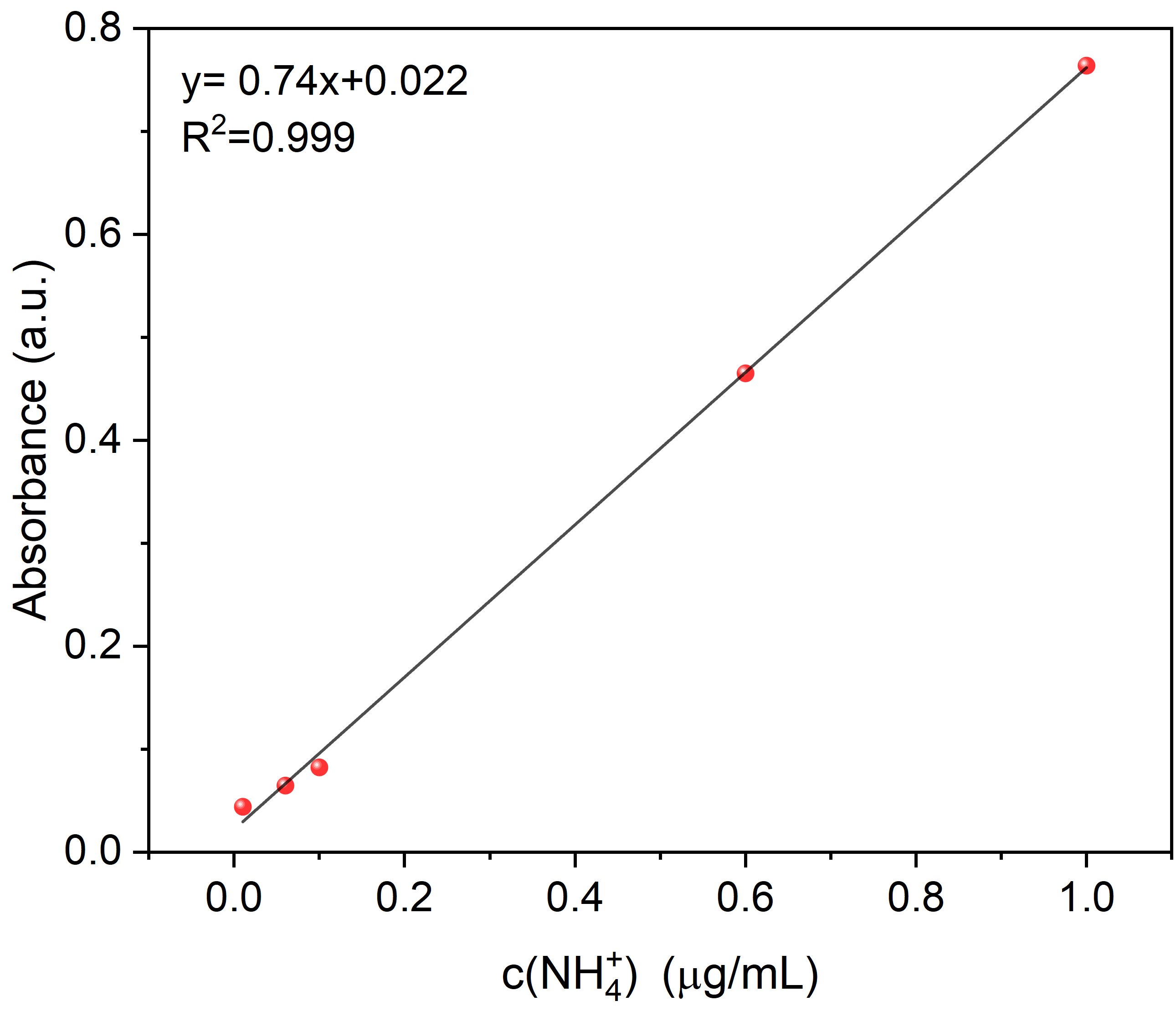


**Figure S12.** Concentration absorbance curve of NH_4_^+^ ions with a series of standard concentration at 0.1 M Na_2_SO_4_ solution.


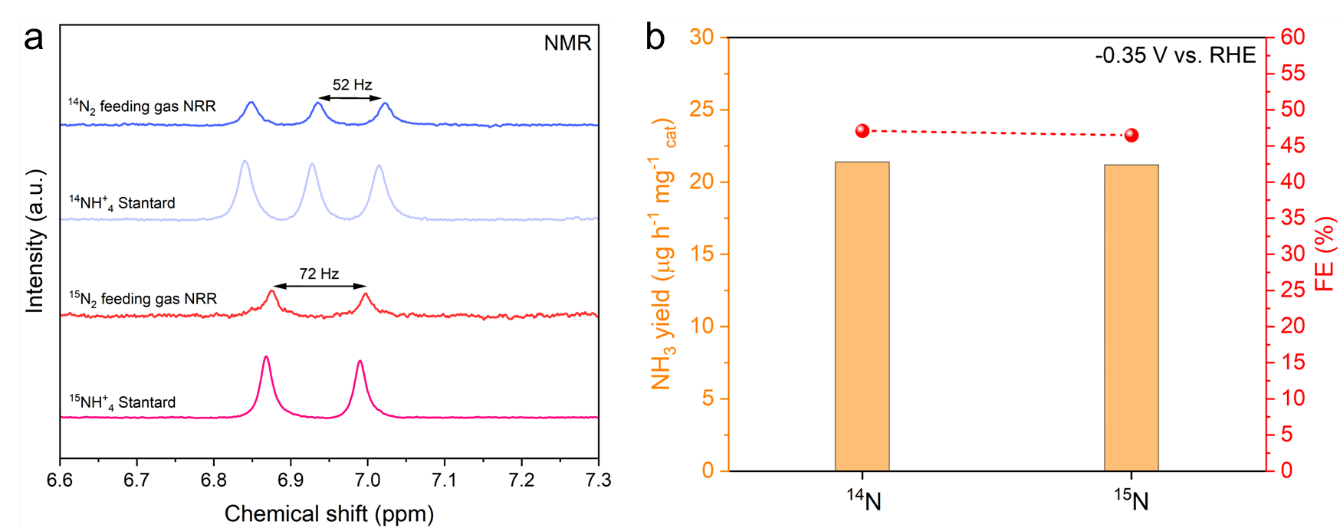


**Figure S13.** a) Comparison of ^1^H NMR spectra of the electrolytes feeding ^15^N_2_/^14^N_2_ and standard samples, b) Comparison of NRR performance using ^14^N_2_ and ^15^N_2_ gases as feedstock gases of Fe_1_-N^S-Ru_1_/Ti_3_C_2_T_x_ in -0.35V vs. RHE.


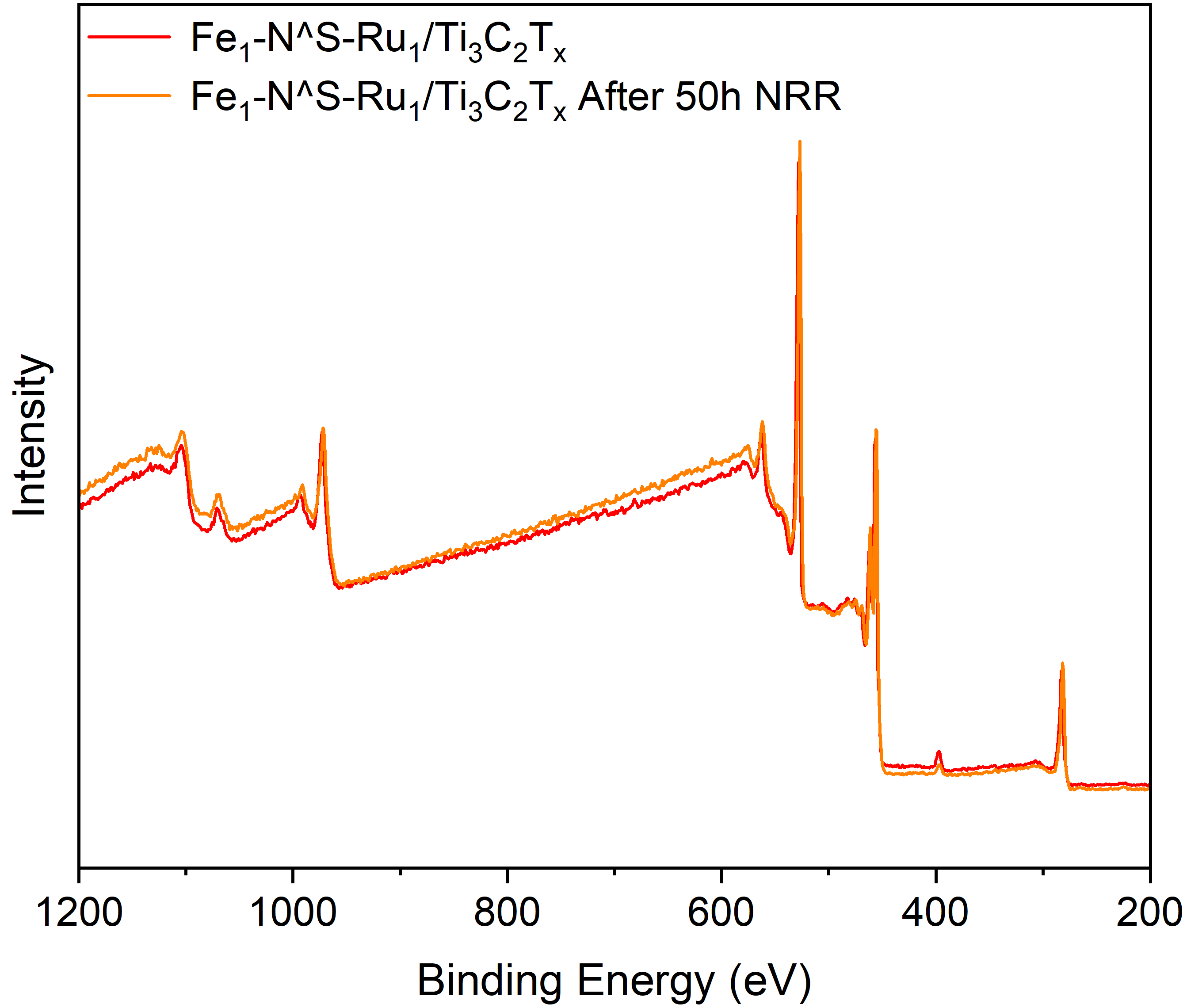


**Figure S14.** XPS spectrum of Fe_1_-N^S-Ru_1_/Ti_3_C_2_T_x_ before and after 50 h chronoamperometry tests.


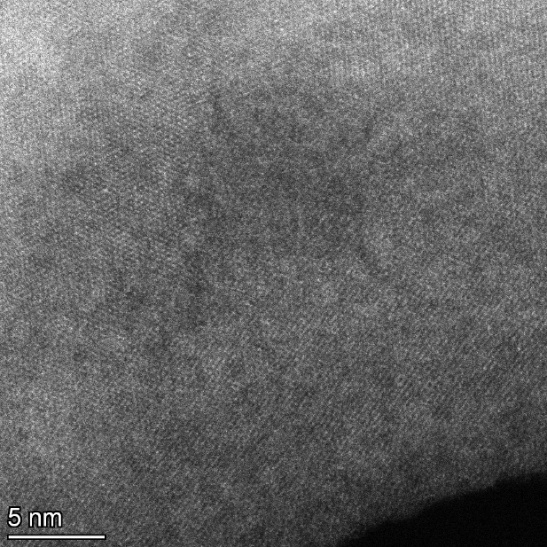


**Figure S15.** AC-HAADF-STEM images of Fe_1_-N^S-Ru_1_/Ti_3_C_2_T_x_ after the 50 h chronoamperometry tests.

**
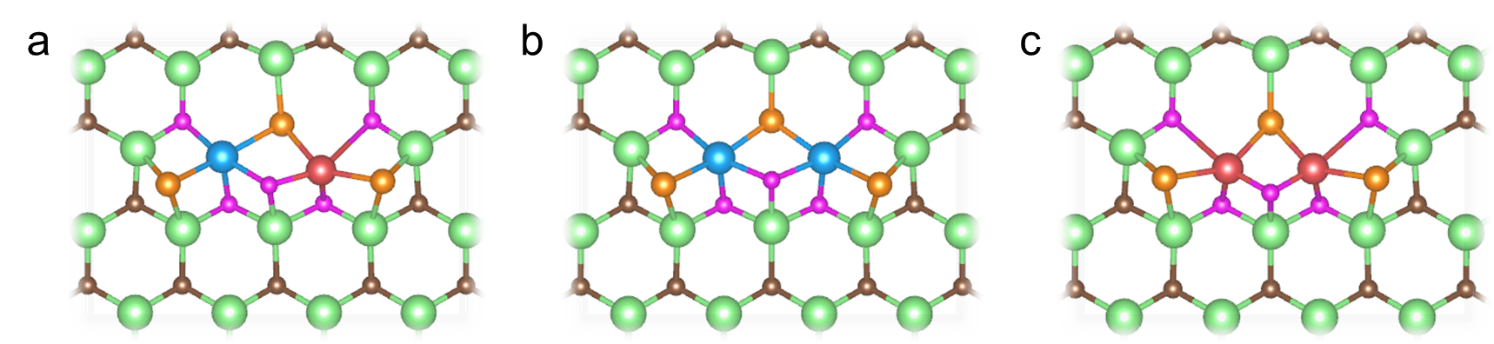
**

**Figure S16.** Structural model of a) Fe_1_-N^S-Ru_1_/Ti_3_C_2_T_x_, b) Ru_1_-N^S-Ru_1_/Ti_3_C_2_T_x_ and c)Fe_1_-N^S-Fe_1_/Ti_3_C_2_T_x_. The blue, red, brown, green, pink, and orange spheres represent Ru, Fe, Ti, C, N, and O elements, respectively.

**
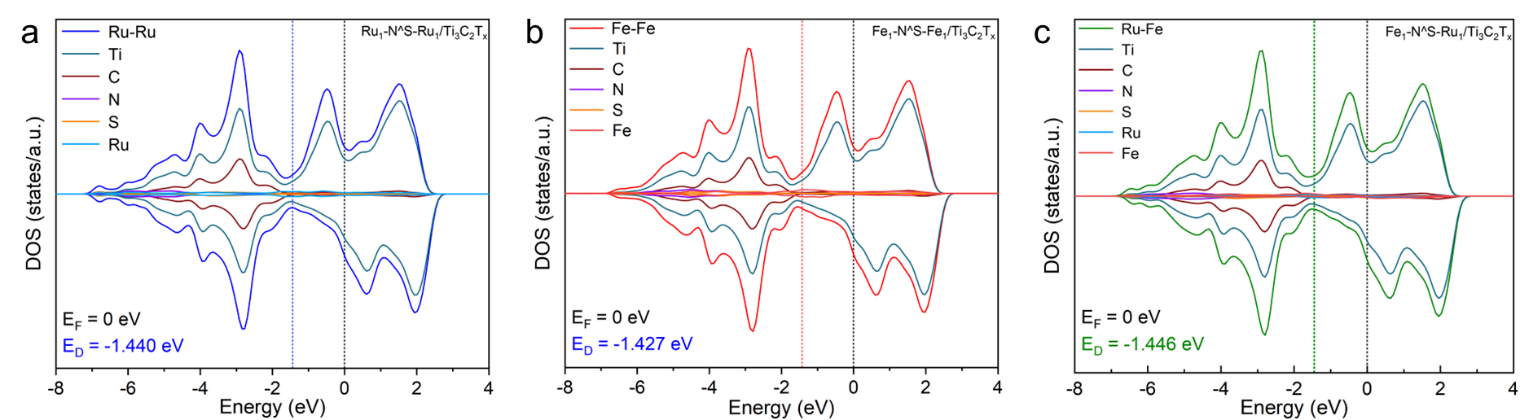
**

**Figure S17.** DOS of a) Ru_1_-N^S-Ru_1_/Ti_3_C_2_T_x_, b) Fe_1_-N^S-Fe_1_/Ti_3_C_2_T_x_ and c) Fe_1_-N^S-Ru_1_/Ti_3_C_2_T_x_.


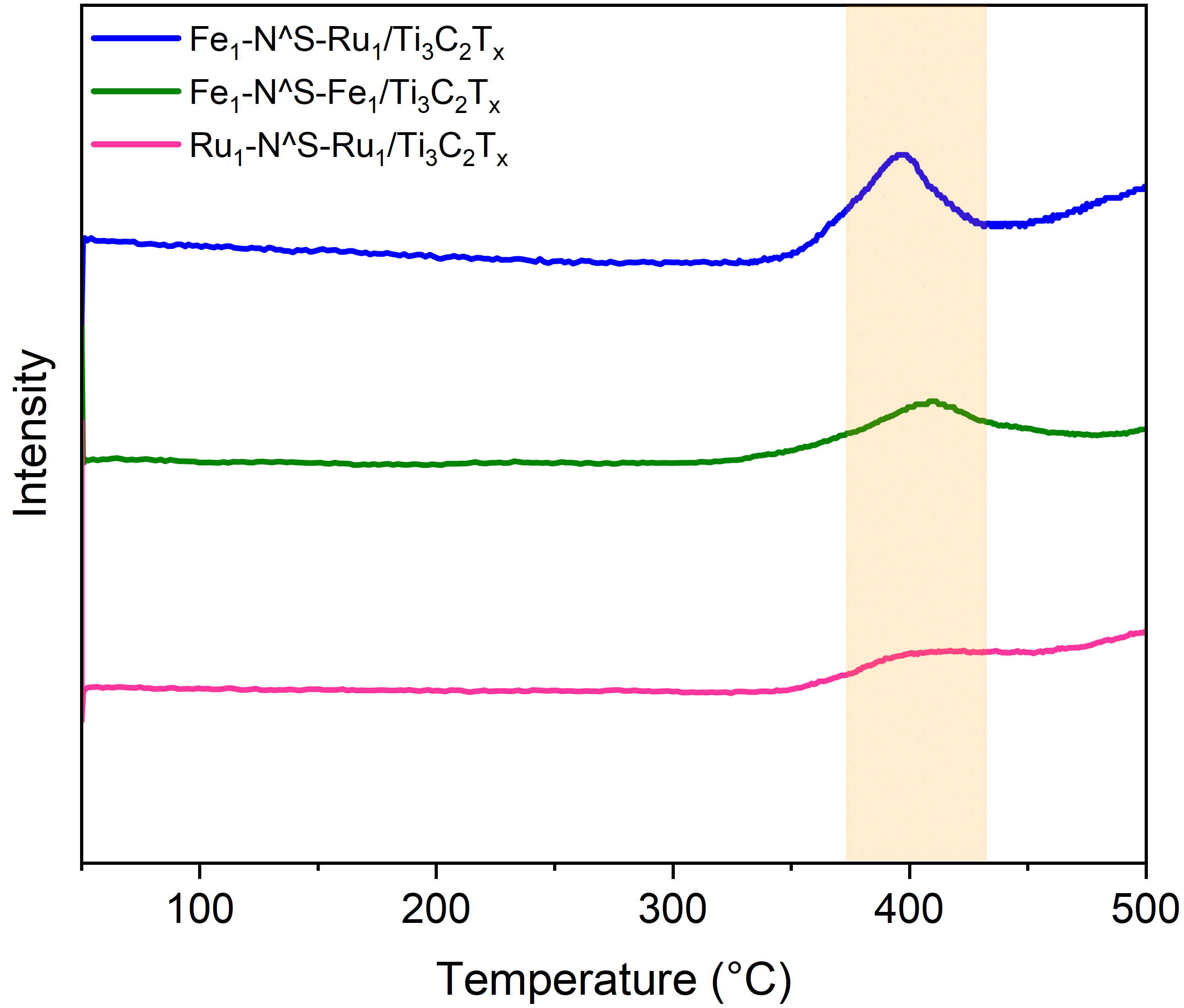


**Figure S18.** N_2_-TPD curves of Fe_1_-N^S-Ru_1_/Ti_3_C_2_T_x_, Fe_1_-N^S-Fe_1_/Ti_3_C_2_T_x_ and Ru_1_-N^S-Fe_1_/Ti_3_C_2_T_x_.


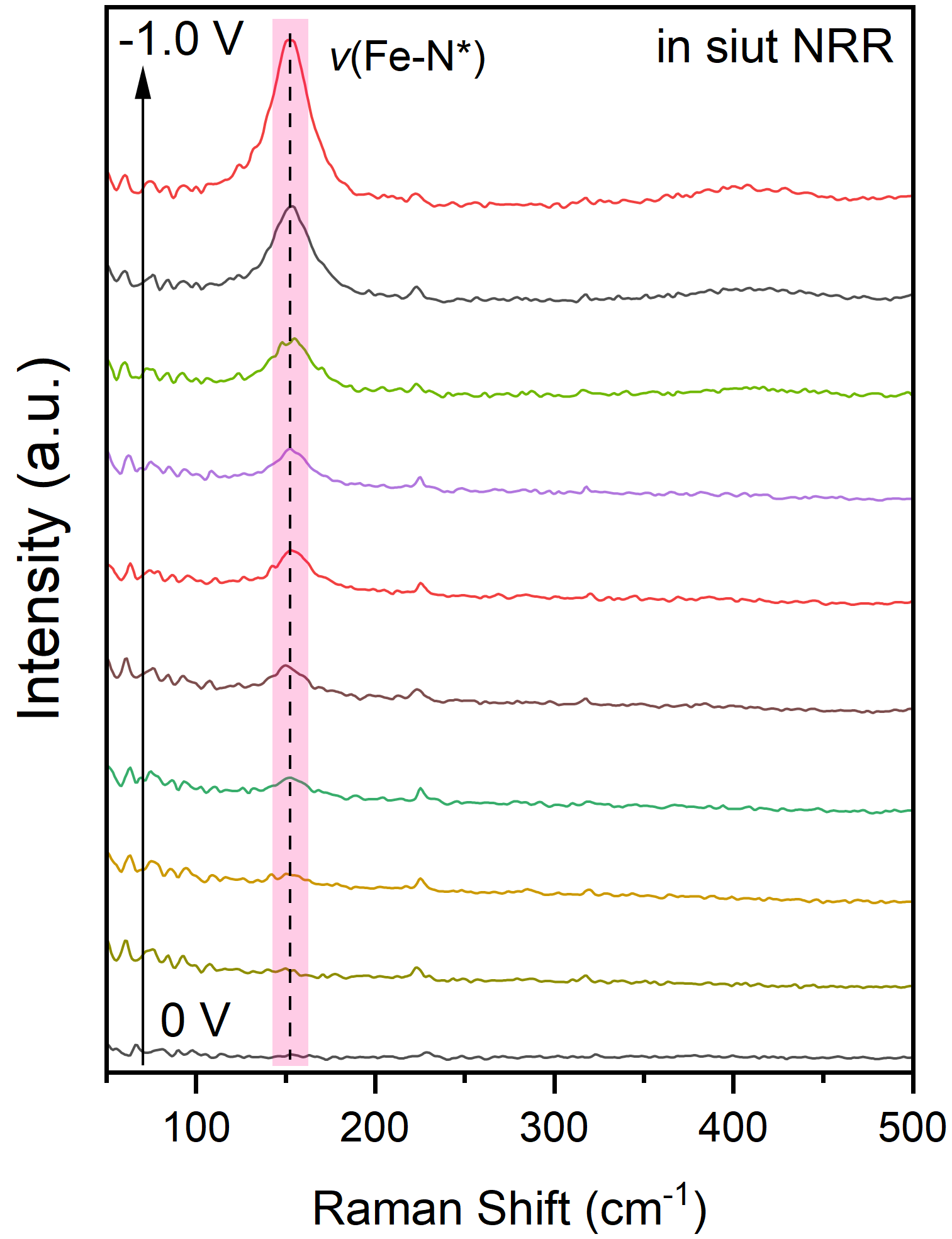


**Figure S19.** In situ Raman tests performed during electrochemical nitrogen reduction process of Fe_1_-N^S-Ru_1_/Ti_3_C_2_T_x_.


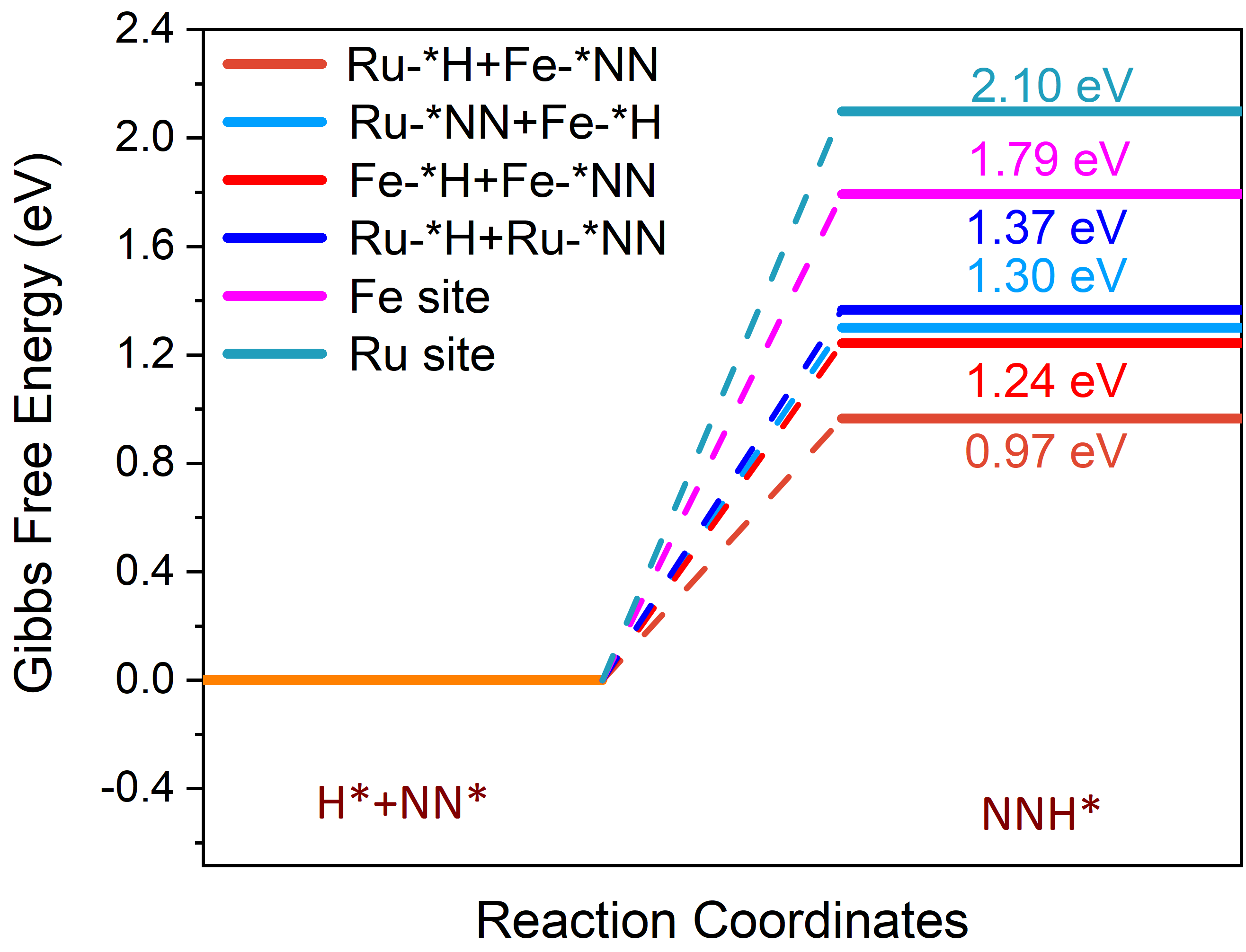


**Figure 20.** Gibbs free energy barrier for the hydrogenation of N_2_* at various metal active sites.


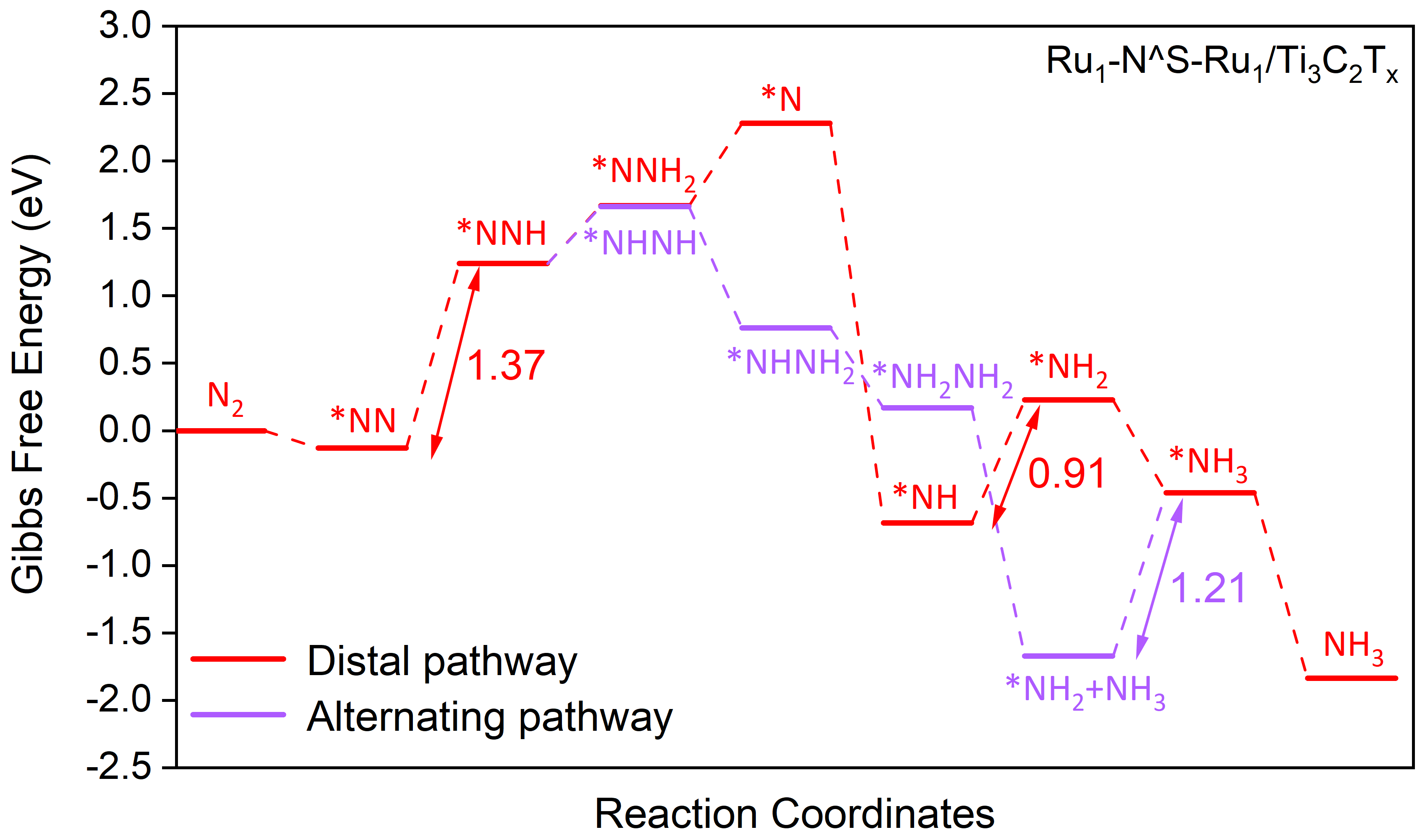


**Figure S21.** Gibbs free energy diagrams for distal and alternating pathway of NRR on Ru_1_-N^S-Ru_1_/Ti_3_C_2_T_x_..

**
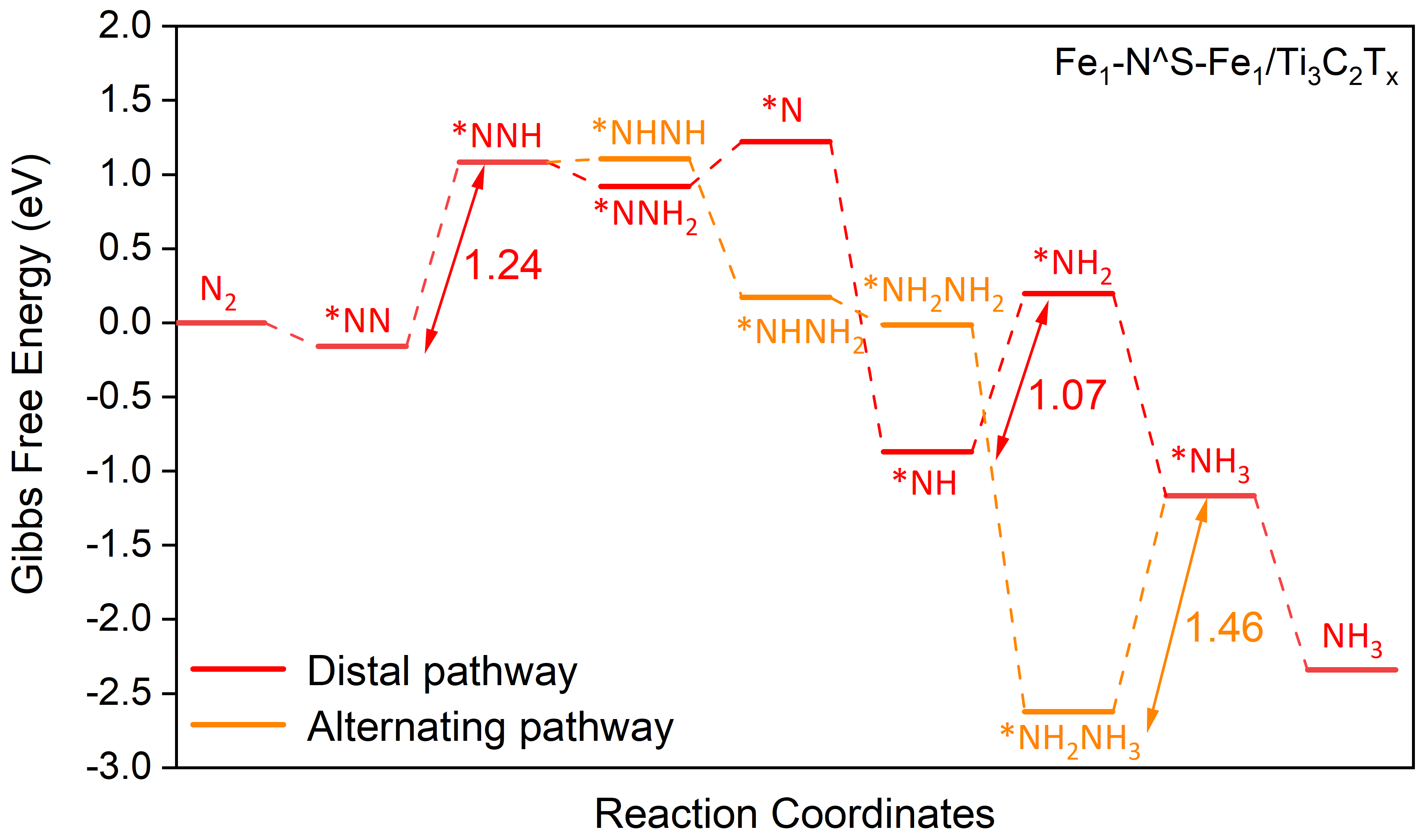
**

**Figure S22.** Gibbs free energy diagrams for distal and alternating pathway of NRR on Fe_1_-N^S-Fe_1_/Ti_3_C_2_T_x_.

**Table S1.** ICP-MS in Ru and Fe mass loading in different catalysts.

^a^Initial sample mass. ^b^Constant volume. ^c^Element concentration of digestion solution.

^d^Sample element mass percent. The concentration of the test solution was diluted tenfold.

**Table S2.** NRR performance comparison with Ti_3_C_2_T_x_-based NRR catalysts.

R range:1.0-3.0 Å; k range: 3.0-14.95 Å.

CN: coordination numbers; R: bond distance; σ^2^: Debye-Waller factors; ΔE_0_: the inner potential correction; R-factor: goodness of fit.

Error bounds that characterize the structural parameters obtained by EXAFS spectroscopy were estimated as CN±20%; R±1%; σ^2^±20%.

**Table S3.** Bader charge analysis data of different samples.

| catalyst | atom | Bader charge | State Value |
| --- | --- | --- | --- |
| Fe_1_-N^S-Ru_1_/Ti_3_C_2_T_x_ | Ru1 | 7.580 | 0.839 |
|  | Fe1 | 7.258 | 1.484 |
| Fe_1_-N^S-Fe_1_/Ti_3_C_2_T_x_ | Fe1 | 7.259 | 1.482 |
|  | Fe2 | 7.260 | 1.480 |
| Ru_1_-N^S-Ru_1_/Ti_3_C_2_T_x_ | Ru1 | 7.552 | 0.897 |
|  | Ru2 | 7.553 | 0.894 |

**Table S4.** N_2_ adsorption energy of different active sites.

| catalyst | active site | N_2_ adsorption energy |
| --- | --- | --- |
| Fe_1_-N^S-Ru_1_/Ti_3_C_2_T_x_ | Ru-Fe Fe | -0.55 |
|  | Ru-Fe Ru | -0.33 |
| Fe_1_-N^S-Fe_1_/Ti_3_C_2_T_x_ | Fe | -0.16 |
| Ru_1_-N^S-Ru_1_/Ti_3_C_2_T_x_ | Ru | -0.13 |

**Table S5.** NRR performance comparison with diatomic catalysts and Ti_3_C_2_T_x_-based NRR catalysts.

| **Catalysts** | **Potential**  **(V vs. RHE)** | **Electrolyte** | **NH_3_ yield rate**  **(μg h^-1^mg^-1^_cat_)** | **FEs**  **(%)** | **Detection method** | **Ref.** |
| --- | --- | --- | --- | --- | --- | --- |
| **Fe_1_-N^S-Ru_1_/Ti_3_C_2_T**_x_ | **-0.25** | **0.1 M Na_2_SO_4_** | **21.5** | **47.1** | **indophenol blue method** | **This**  **work** |
| Al_2_-NC | -0.60 | 0.1 M Na_2_SO_4_ | 29.2 | 16.6 | indophenol blue method | [6] |
| FeMoNC | -0.25 | 0.1 M KOH | 54.40 | 18.0 | indophenol blue method | [7] |
| Ru Sas/GDY/G | -0.1 | 0.1 M Na_2_SO_4_ | 56.8 | 37.6 | indophenol blue method | [8] |
| Bi_2_S_3-x_/Ti_3_C_2_T_x_ | -0.6 | 0.1 M Li_2_SO_4_ | 68.3 | 22.5 | indophenol blue method | [9] |
| Ti_3_C_2_T_x_  QDs | -0.5 | 0.1 M HCl | 62.94 | 13.30 | indophenol blue method | [10] |
| MXene/TiFeO_x_-700 | -0.2 | 0.05 M H_2_SO_4_ | 21.9 | 21.9 | indophenol blue method | [11] |
| Ti_3_C_2_T_x_-medium F | -0.7 | 0.01 M Na_2_SO_4_ | 1.72 | 7.38 | indophenol blue method | [12] |
| Ti_3_C_2_T_x_ | -0.40 | 0.1 M HCl | 20.4 | 9.3 | indophenol blue method | [13] |
| TiO_2_/Ti_3_C_2_T_x_ | -0.55 | 0.1 M HCl | 32.2 | 16.07 | indophenol blue method | [14] |

***Supplementary References***

[1] Song, W.; Li, C.; Ma, P.; Liu, X.; Guo, Y.; Jia, M.; Zhang, W.; He, C. Computational Screening of Transition Metal Atom Doped C_3_N as Electrocatalysts for Nitrogen Fixation. *Mol. Catal.* **2023**, *535*, 112888.

[2] Mao, X.; Gu, Z.; Yan, C.; Du, A. Unlocking the Potential of Ruthenium Catalysts for Nitrogen Fixation with Subsurface Oxygen. *J. Mater. Chem. A* **2021**, *9* (10), 6575–6582.

[3] Liao, X.; Lu, R.; Xia, L.; Liu, Q.; Wang, H.; Zhao, K.; Wang, Z.; Zhao, Y. Density Functional Theory for Electrocatalysis. *Energy Environ. Mater.* **2022**, *5* (1), 157–185.

[4] Kong, W.; Xu, J.; Tong, Y.; Ding, Y.; Wang, J.; Li, B.; Wei, X.; Zhao, L. Construction of Dual Active Sites for Efficient Alkaline Hydrogen Evolution: Single-Metal-Atoms Supported on BC2N Monolayers. *Phys. Chem. Chem. Phys.* **2022**, *24* (47), 29141–29150.

[5] Wu, Z.-Y.; Karamad, M.; Yong, X.; Huang, Q.; Cullen, D. A.; Zhu, P.; Xia, C.; Xiao, Q.; Shakouri, M.; Chen, F.-Y.; Kim, J. Y. (Timothy); Xia, Y.; Heck, K.; Hu, Y.; Wong, M. S.; Li, Q.; Gates, I.; Siahrostami, S.; Wang, H. Electrochemical Ammonia Synthesis via Nitrate Reduction on Fe Single Atom Catalyst. *Nat. Commun* **2021**, *12* (1), 2870.

[6] Biswas, S.; Zhou, J.; Chen, X.; Chi, C.; Pan, Y.; Cui, P.; Li, J.; Liu, C.; Xia, X. Synergistic Al−Al Dual‐Atomic Site for Efficient Artificial Nitrogen Fixation. Angew Chem Int Ed. 2024, 63 (24), e202405493.

[7] Li, R.; Ma, R.; Zhang, L.-L.; Ma, W.; Shao, G.; Zhang, X.; Tian, Y.; Jiao, M.; Zhou, Z. High-Efficiency Electrochemical Ammonia Synthesis at Co-Catalytic Fe-Mo Dual-Atom Sites. ACS Nano 2025, 19 (18), 17686-17697.

[8] Feng, X.; Liu, J.; Chen, L.; Kong, Y.; Zhang, Z.; Zhang, Z.; Wang, D.; Liu, W.; Li, S.; Tong, L.; Zhang, J. Hydrogen Radical-Induced Electrocatalytic N2 Reduction at a Low Potential. J. Am. Chem. Soc. 2023, 145 (18), 10259-10267.

[9] Luo, Y.; Shen, P.; Li, X.; Guo, Y.; Chu, K. Sulfur-Deficient Bi2S3−x Synergistically Coupling Ti3C2Tx-MXene for Boosting Electrocatalytic N2 Reduction. Nano Res. 2022, 15 (5), 3991-3999.

[10] Jin, Z.; Liu, C.; Liu, Z.; Han, J.; Fang, Y.; Han, Y.; Niu, Y.; Wu, Y.; Sun, C.; Xu, Y. Rational Design of Hydroxyl-Rich Ti3C2Tx MXene Quantum Dots for High-Performance Electrochemical N2 Reduction. Adv. Energy Mater. 2020, 10 (22), 2000797.

[11] Guo, Y.; Wang, T.; Yang, Q.; Li, X.; Li, H.; Wang, Y.; Jiao, T.; Huang, Z.; Dong, B.; Zhang, W.; Fan, J.; Zhi, C. Highly Efficient Electrochemical Reduction of Nitrogen to Ammonia on Surface Termination Modified Ti3C2Tx MXene Nanosheets. ACS Nano 2020, 14 (7), 9089-9097.

[12] Ding, Y.; Zhang, J.; Guan, A.; Wang, Q.; Li, S.; Al-Enizi, A. M.; Qian, L.; Zhang, L.; Zheng, G. Promoting N2 Electroreduction to Ammonia by Fluorine-Terminating Ti3C2Tx MXene. Nano Converg. 2021, 8 (1), 14.

[13] Zhao, J.; Zhang, L.; Xie, X.-Y.; Li, X.; Ma, Y.; Liu, Q.; Fang, W.-H.; Shi, X.; Cui, G.; Sun, X. Ti_3_C_2_T_x_ (T = F, OH) MXene Nanosheets: Conductive 2D Catalysts for Ambient Electro hydrogenation of N2 to NH3. J. Mater. Chem. A 2018, 6 (47), 24031-24035.

[14] Fang, Y.; Liu, Z.; Han, J.; Jin, Z.; Han, Y.; Wang, F.; Niu, Y.; Wu, Y.; Xu, Y. High-Performance Electrocatalytic Conversion of N2 to NH3 Using Oxygen-Vacancy-Rich TiO2 In Situ Grown on Ti3C2Tx MXene. Adv. Energy Mater. 2019, 9 (16), 1803406.
